# Supplementary figures and images for: Germline-encoded specificities and the predictability of the B cell response
Source: PLoS Pathog. 2023 Aug 25;19(8):e1011603. doi: 10.1371/journal.ppat.1011603 (PMC10484431; doi:10.1371/journal.ppat.1011603)

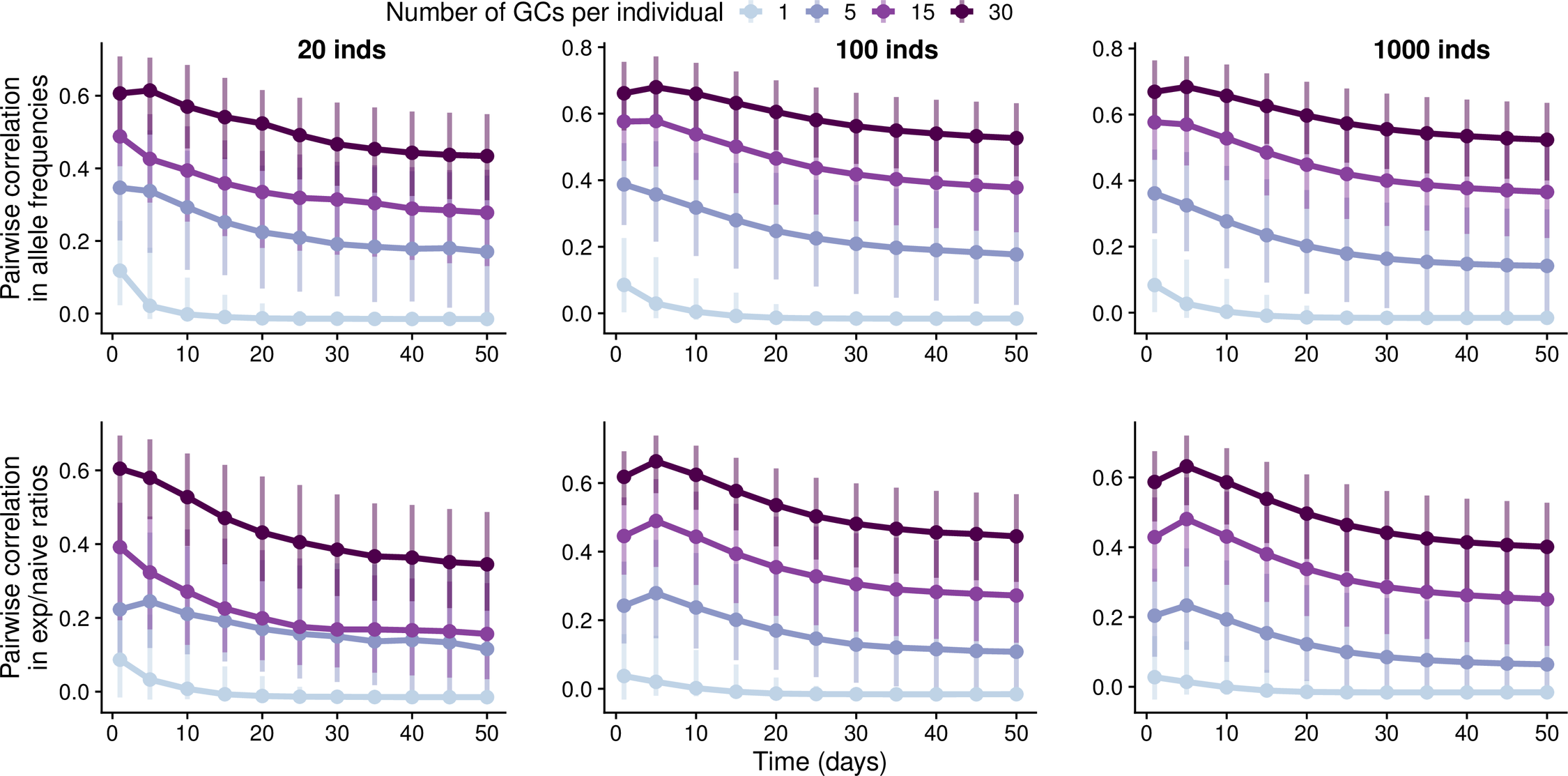

Supplement: S1 Fig — We measured germline allele frequencies across germinal centers in each individual and computed the ratio between these frequencies and the allele frequencies in the naive repertoire. Points and vertical bars represent the median and the 1st and 4th quartiles of these correlations across all pairs (i.e., the bars represent true variation in simulated outcomes and not the uncertainty in the estimate of the median). We let 5 germline alleles have the mean of their naive affinity distribution increased by s = 1.5 relative to the baseline. We set the mutation rate to 0.01 mutations per B cell division and β = 4. Other parameters were set to the default values in Table 1. (TIF) [file ppat.1011603.s001.tif]

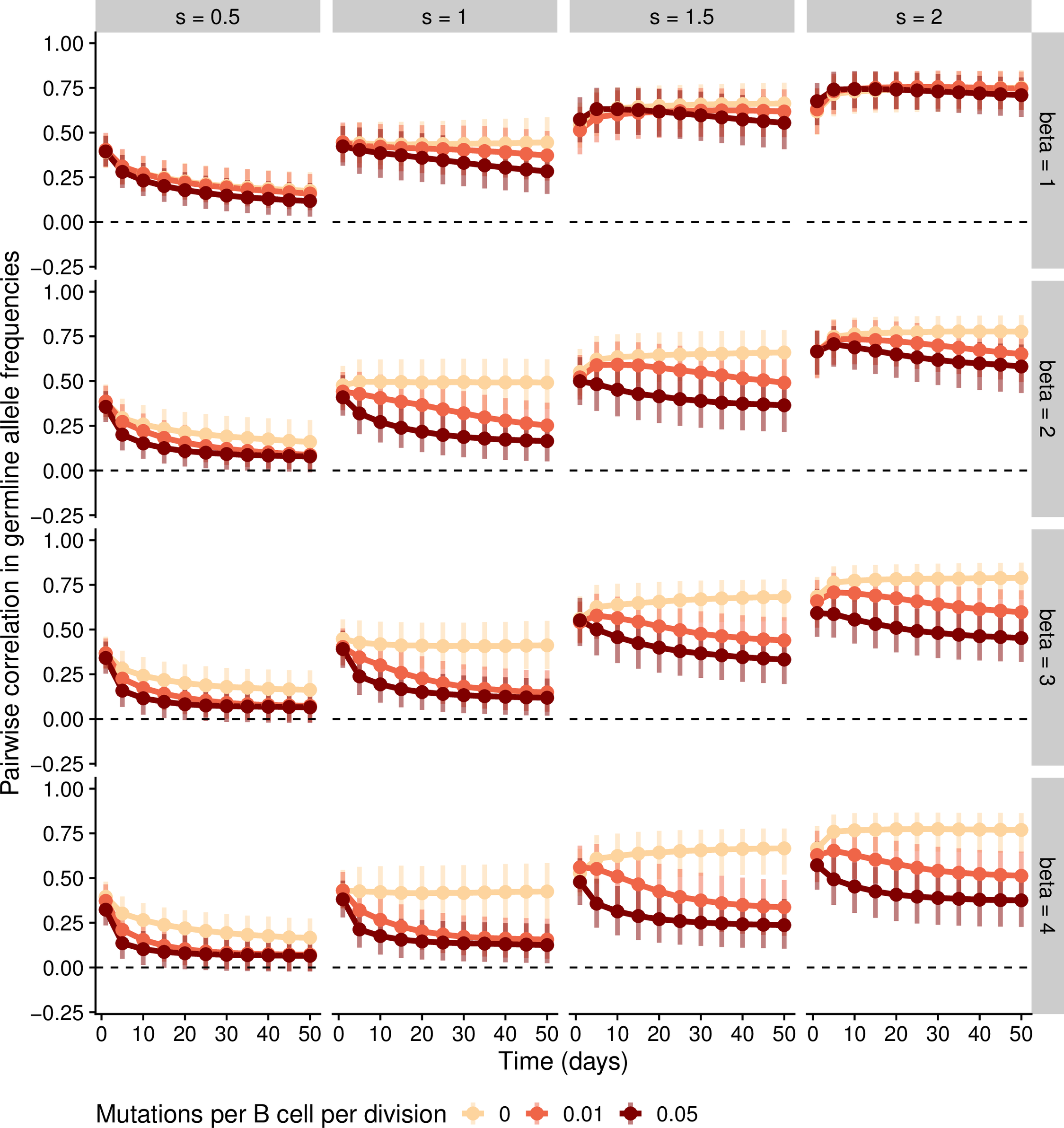

Supplement: S2 Fig — For each parameter combination, we simulated 100 individuals with varying numbers of germinal centers. We measured germline allele frequencies across germinal centers in each individual and computed the correlation in those frequencies between all pairs of individuals. Points and vertical bars represent the median and the 1st and 4th quartiles of these correlations across all pairs (i.e., the bars represent true variation in simulated outcomes and not the uncertainty in the estimate of the median). For these simulations, we assumed 15 germinal centers per individual. Other parameter values are as in Table 1. (TIF) [file ppat.1011603.s002.tif]

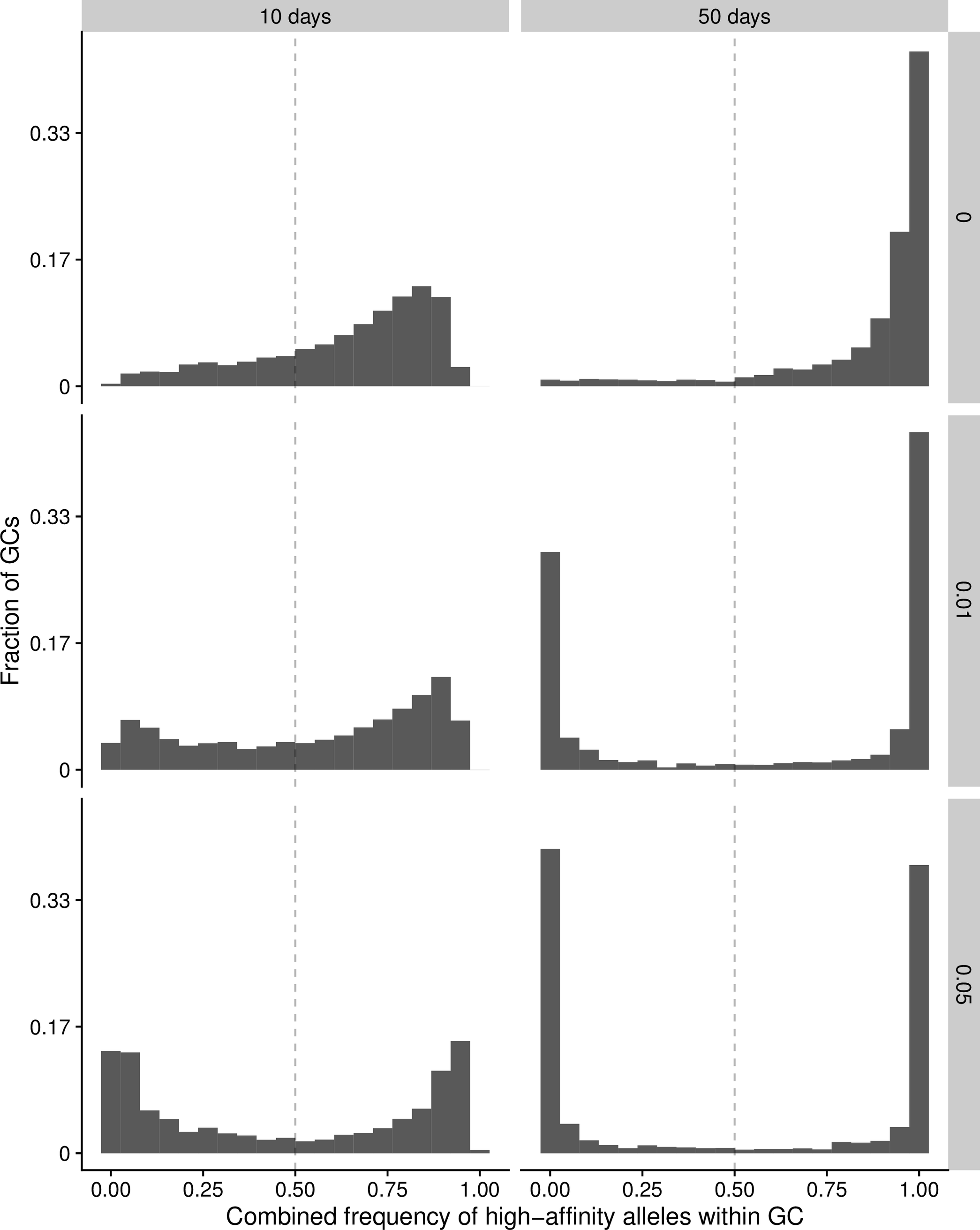

Supplement: S3 Fig — Columns show the distribution across germinal centers early (10 days) and late (50 days) in the response. Rows show different somatic hypermutation rates (mutations per B cell per division). For each row, we simulated 100 individuals, each with 30 germinal centers. The same five alleles in all individuals were chosen to have their average naive affinity increased by s = 2. We set β = 4 and other parameters to the values in Table 1. (TIF) [file ppat.1011603.s003.tif]

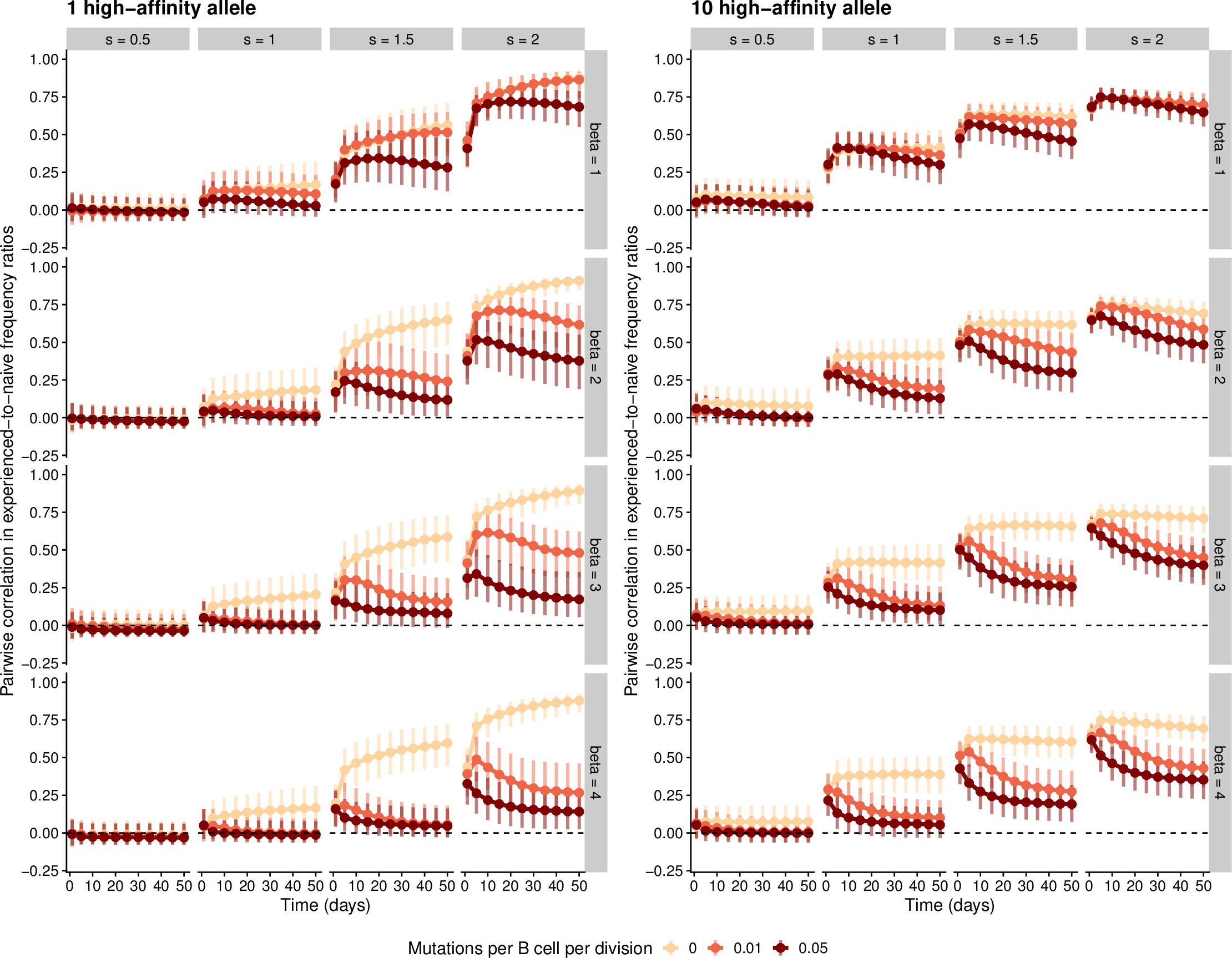

Supplement: S4 Fig — For each parameter combination, we simulated 100 individuals with varying numbers of germinal centers. We measured germline allele frequencies across germinal centers in each individual and computed the ratio between these frequencies and the allele frequencies in the naive repertoire. We then computed the correlation in these experienced-to-naive-ratios between all pairs of individuals. Points and vertical bars represent the median and the 1st and 4th quartiles of these correlations across all pairs (i.e., the bars represent true variation in simulated outcomes and not the uncertainty in the estimate of the median). For these simulations, we assumed 15 germinal centers per individual. Other parameter values are as in Table 1. (TIF) [file ppat.1011603.s004.tif]

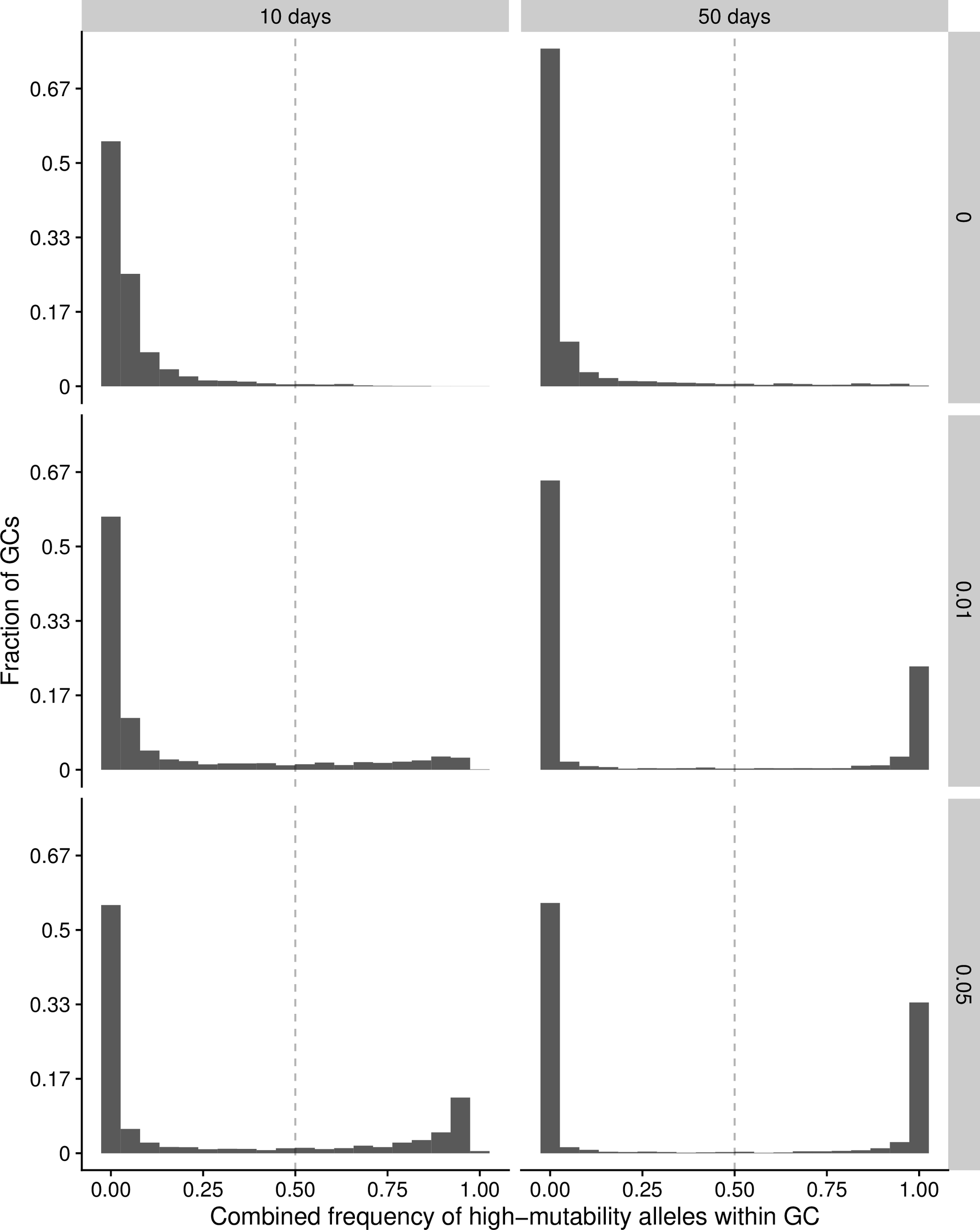

Supplement: S5 Fig — Columns show the distribution across germinal centers early (10 days) and late (50 days) in the response. Rows show different baseline mutation rates (affinity-changing mutations per B cell per division). For each row, we simulated 100 individuals, each with 30 germinal centers. The same five alleles in all individuals were chosen to have their baseline mutation rate increased by γ = 6. We set β = 4 and other parameters to the values in Table 1. (TIF) [file ppat.1011603.s005.tif]

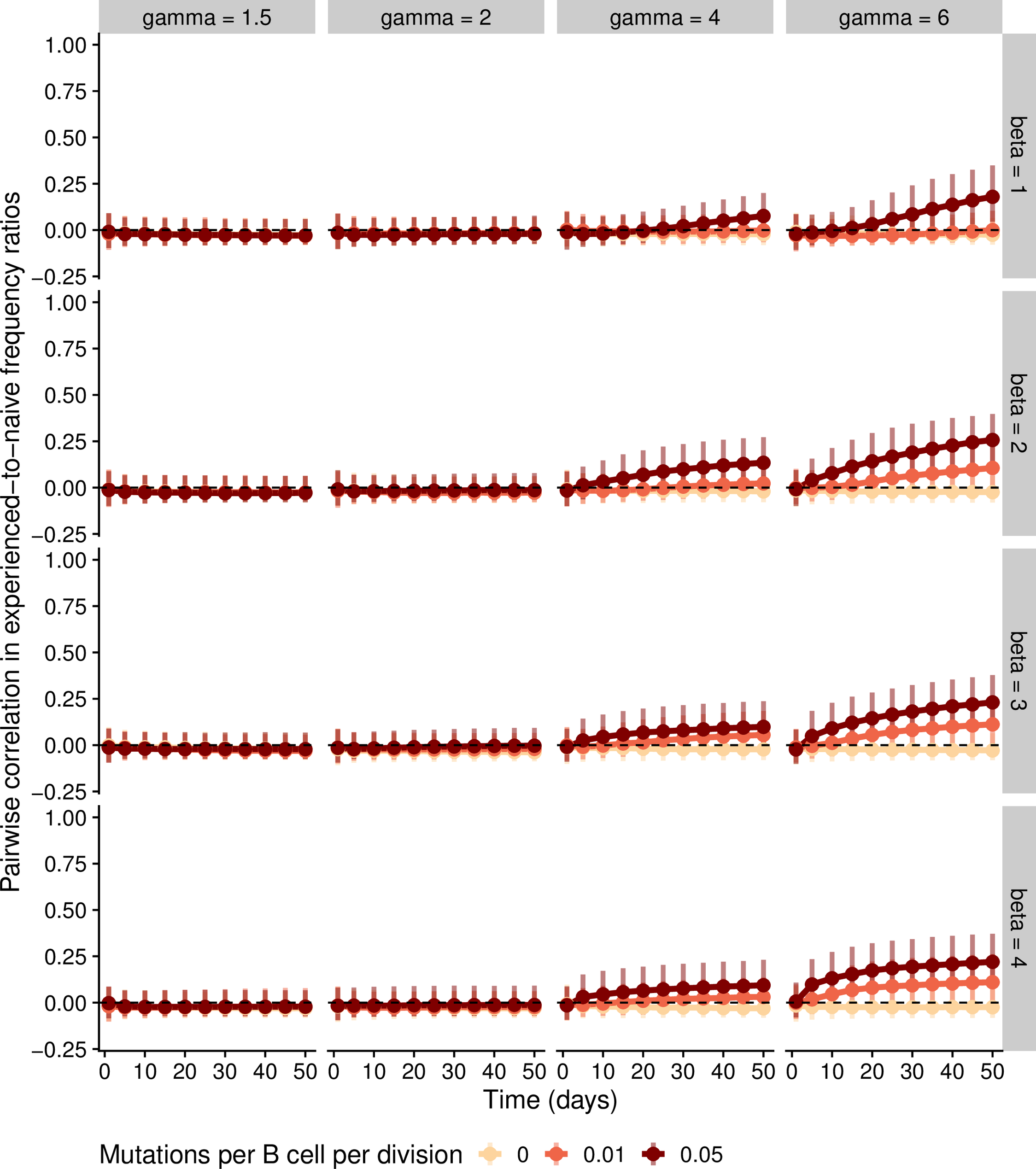

Supplement: S6 Fig — For each parameter combination, we simulated 100 individuals with varying numbers of germinal centers. We measured germline allele frequencies across germinal centers in each individual and computed the ratio between these frequencies and the allele frequencies in the naive repertoire. We then computed the correlation in these experienced-to-naive-ratios between all pairs of individuals. Points and vertical bars represent the median and the 1st and 4th quartiles of these correlations across all pairs (i.e., the bars represent true variation in simulated outcomes and not the uncertainty in the estimate of the median). For these simulations, we assumed 15 germinal centers per individual. γ represents the factor by which the baseline mutation rate is multiplied in high-mutation alleles, while β represents the standard deviation of mutation effect size on affinity. Other parameter values are as in Table 1. (TIF) [file ppat.1011603.s006.tif]

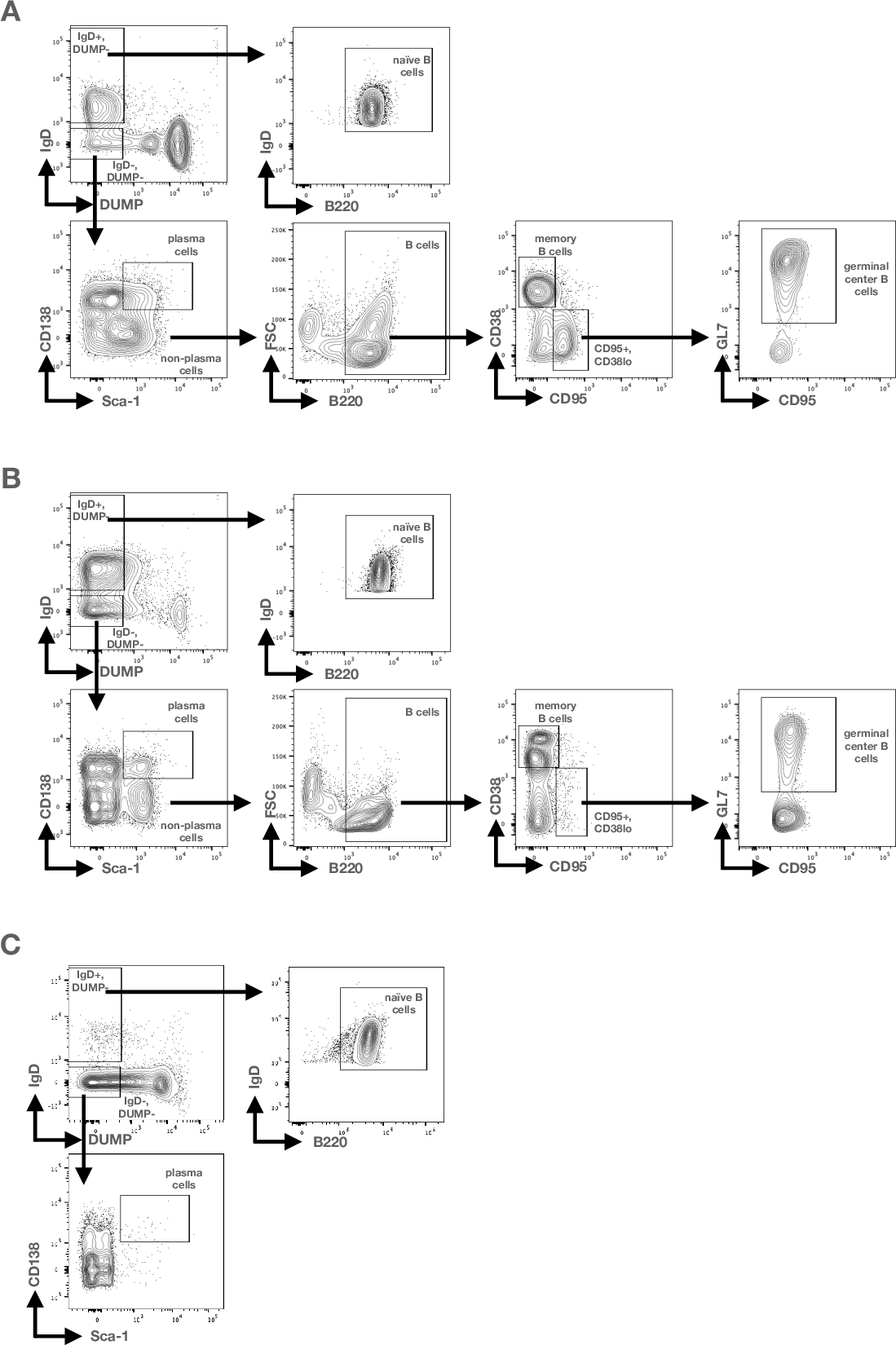

Supplement: S7 Fig — Representative plots for gating strategy for mediastinal lymph nodes (A), spleen (B), and bone marrow (C). Debris and doublets were first gated out (not shown), followed by exclusion of cells expressing CD4, CD8, TER-119, and/or F4/80 (DUMP). Lymph node (A) and spleen (B) IgD+DUMP- cells that were positive for B220 were sorted as naïve B cells. IgD-DUMP- cells that were Sca-1hiCD138hi were sorted as plasma cells. Sca-1lo/-CD138lo/- cells that were also B220+ were further gated and sorted as memory cells (CD95-CD38hi) or germinal center B cells (CD95+CD38lowGL7+). From bone marrow, we only sorted naïve B cells (IgD+DUMP-B220+) and plasma cells (IgD-DUMP- Sca-1hiCD138hi) (C). These representative plots show tissue from one mouse 56 days after primary infection. (TIF) [file ppat.1011603.s007.tif]

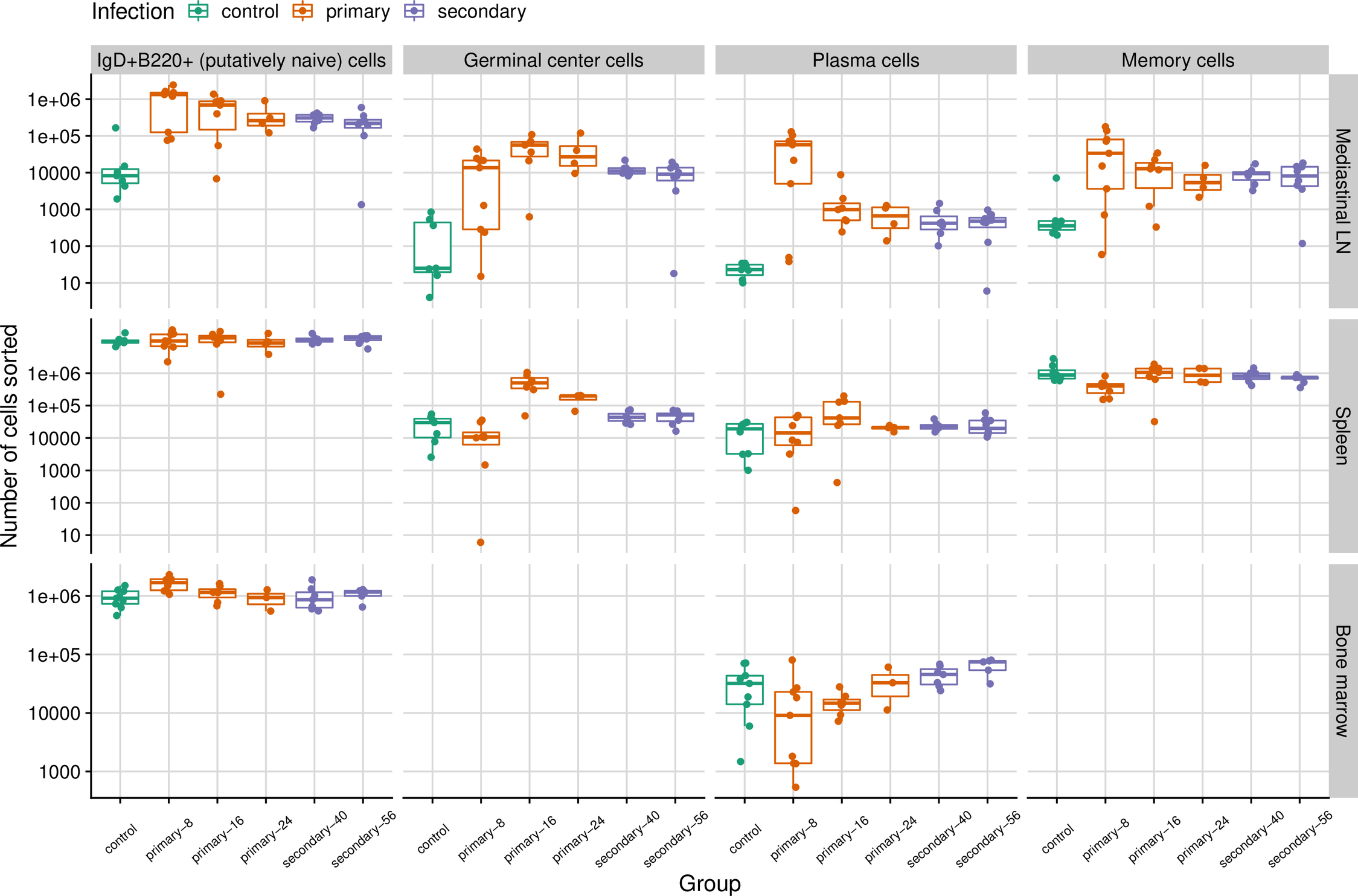

Supplement: S8 Fig — Infected mice were subject to one or two infections and sacrificed at 8, 16, 24, 40 or 56 days after primary infection. Mice from the last two time points were given a second infection 32 days after the first one. (TIF) [file ppat.1011603.s008.tif]

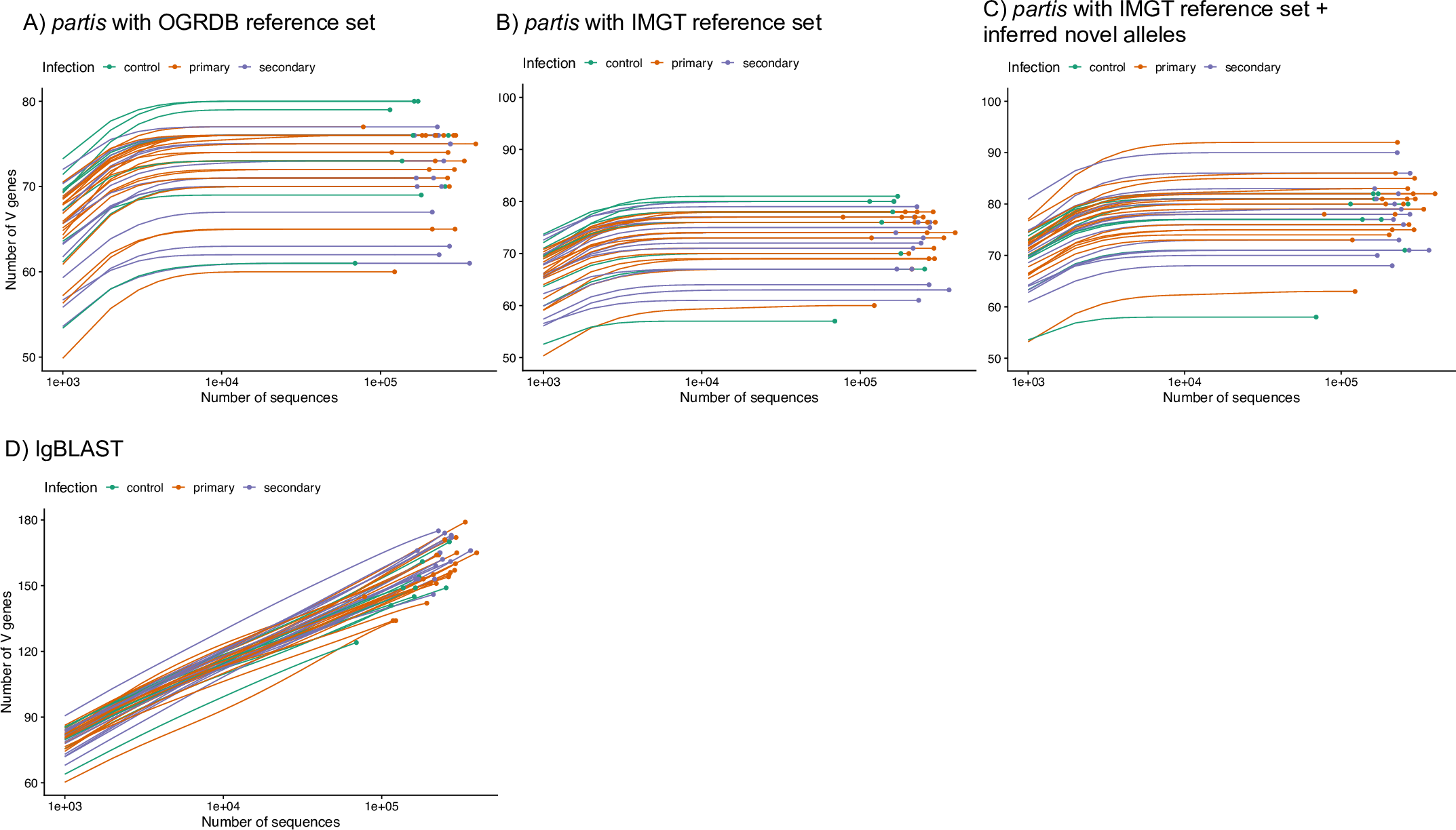

Supplement: S9 Fig — Each circle represents the observed value for an individual mouse, with the associated rarefaction curve indicating the expected number of alleles if only a random subset of the mouse’s sequences had been sampled. (TIF) [file ppat.1011603.s009.tif]

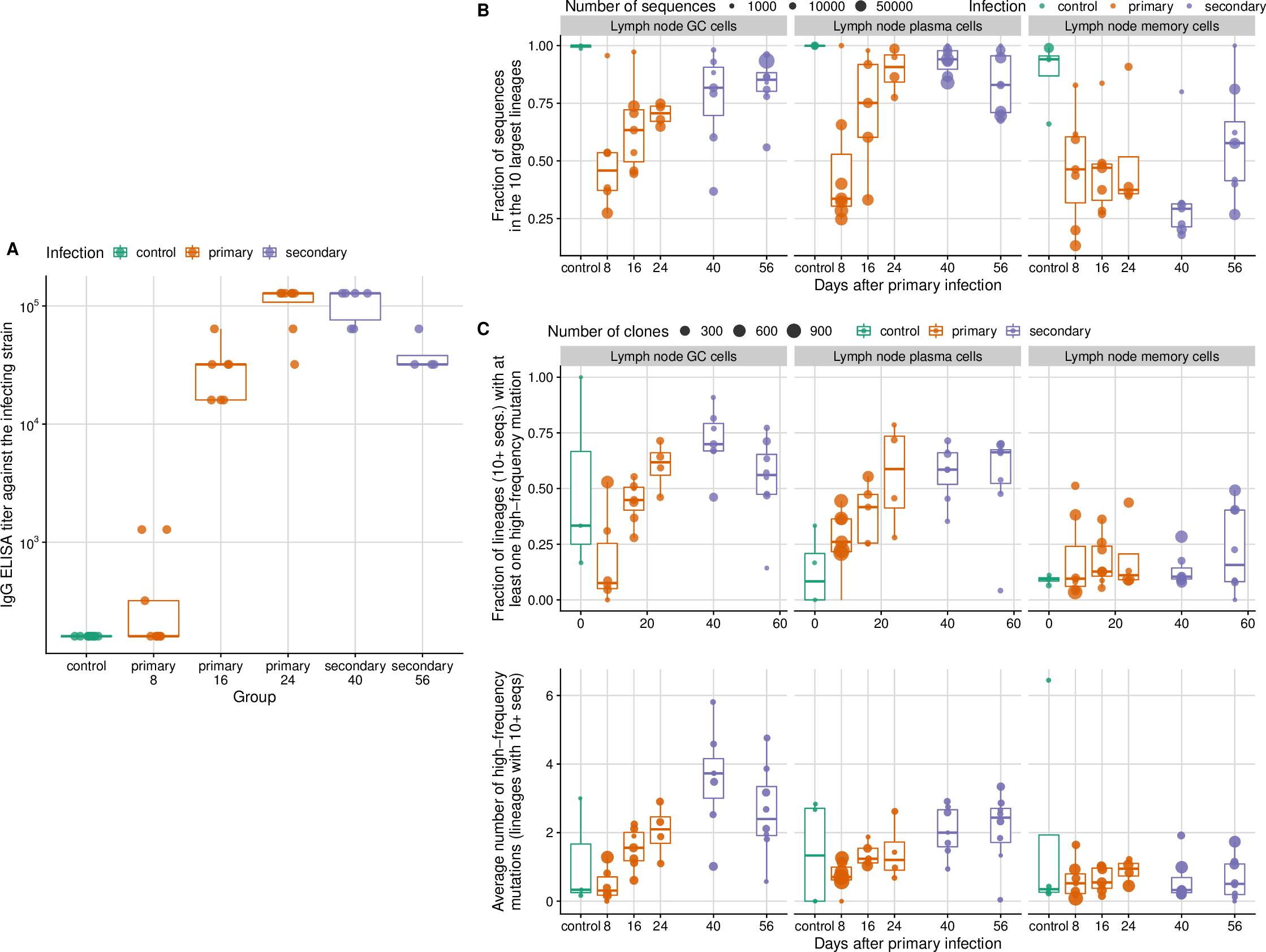

Supplement: S10 Fig — (A) Serum antibody titers against the infecting strain measured by ELISA. (B) Total fraction of reads in influenza-induced populations represented by the ten largest B lineages in each mouse. The ten largest lineages were chosen based on the number of reads each lineage had in the respective cell type in the lymph node (not the total number of reads each lineage had across all tissue and cell types). (C) Fraction of lineages with at least one amino acid mutation at frequency 50% of higher in the lineage (top panel), and the average number of such high-frequency mutations per lineage within each mouse (bottom panel). Mutation frequencies in each lineage were calculated relative to the lineage’s number of reads in the respective tissue and cell type combinations. For these calculations, only lineages with at least 10 reads were considered. (TIF) [file ppat.1011603.s010.tif]

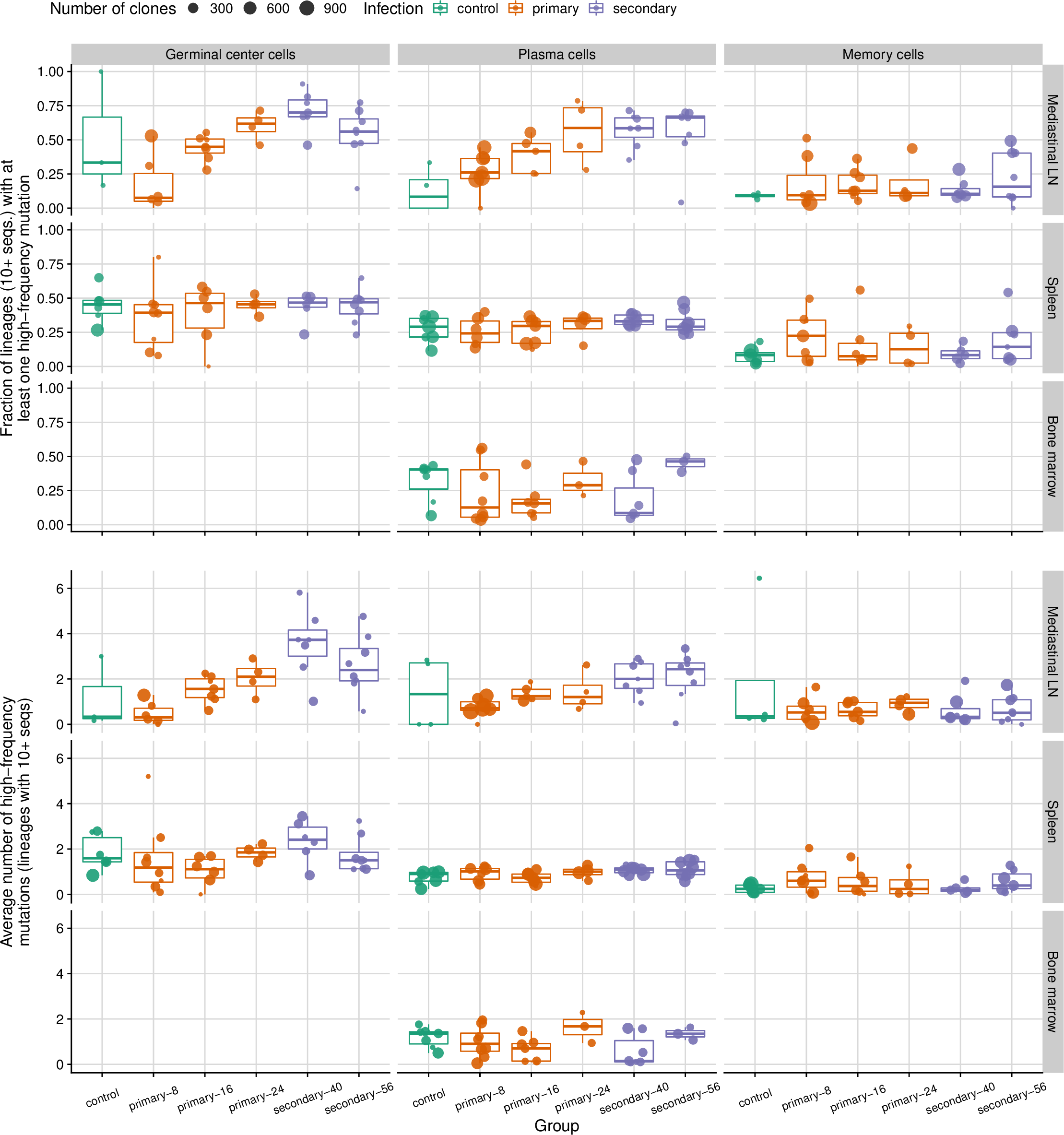

Supplement: S11 Fig — Fraction of clones with at least one amino acid mutation at frequency 50% of higher (top panel) and the average number of such high-frequency mutations per clone (bottom panel) for different cell types and tissues. Mutation frequencies in each clone were calculated relative to the clone’s number of reads in the respective tissue and cell type combinations (not the total number of reads in the clone across all subtypes and tissues). For each combination of cell type and tissue, each point corresponds to a mouse. Only clones with at least ten reads were considered. (TIF) [file ppat.1011603.s011.tif]

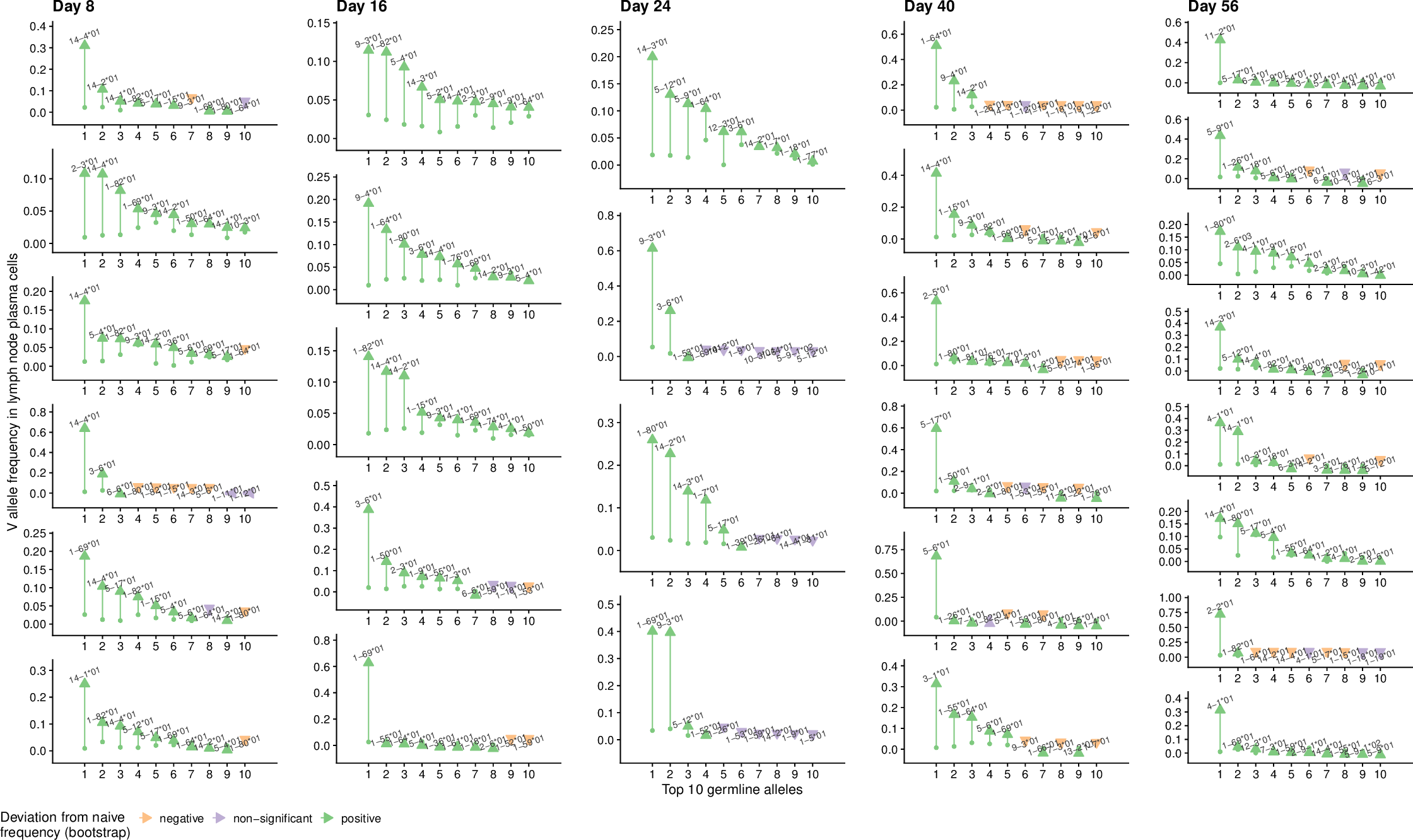

Supplement: S12 Fig — Each panel represents an individual mouse. The arrows go from each allele’s frequency in the naive repertoire to its frequency in lymph node plasma cells. Mouse 40-7, which was sacrificed 8 days after the secondary infection, was considered a day-8 primary-infection mouse because it showed no signs of infection after the first inoculation and had ELISA titers similar to those of day-8 infected mice. (TIF) [file ppat.1011603.s012.tif]

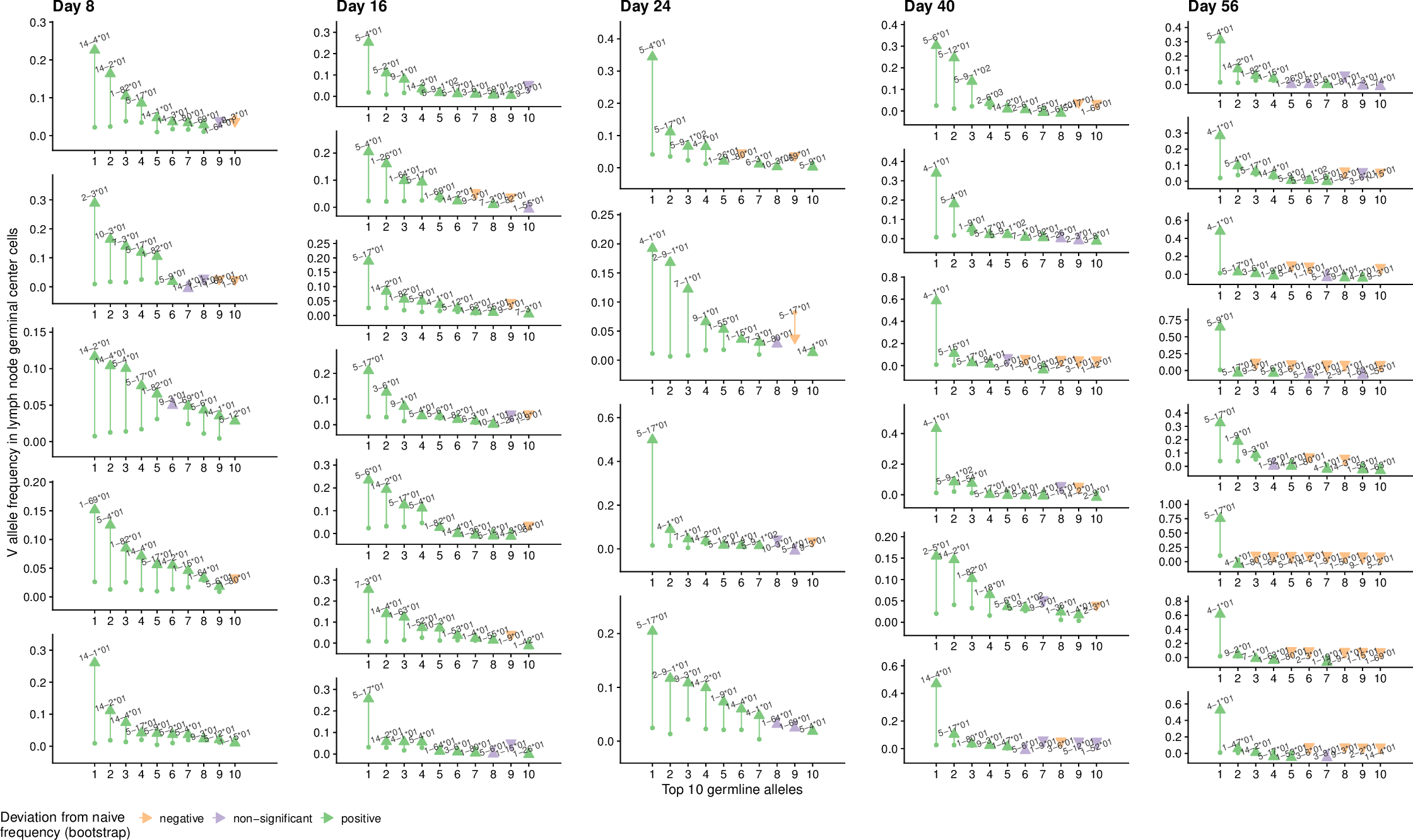

Supplement: S13 Fig — Each panel represents an individual mouse. The arrows go from each allele’s frequency in the naive repertoire to its frequency in lymph node plasma cells. Mouse 40-7, which was sacrificed 8 days after the secondary infection, was considered a day-8 primary-infection mouse because it showed no signs of infection after the first inoculation and had ELISA titers similar to those of day-8 infected mice. (TIF) [file ppat.1011603.s013.tif]

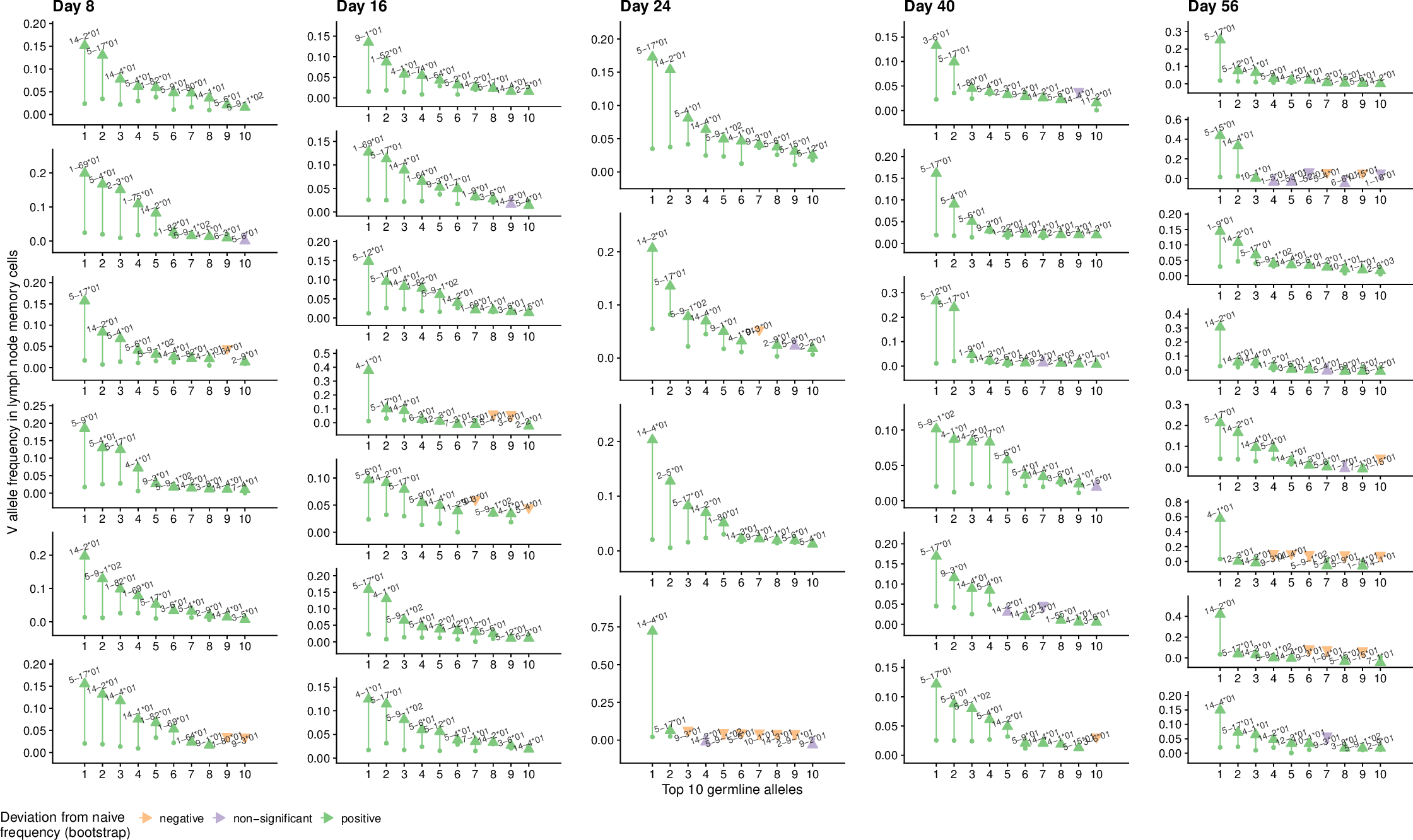

Supplement: S14 Fig — Each panel represents an individual mouse. The arrows go from each allele’s frequency in the naive repertoire to its frequency in lymph node plasma cells. Mouse 40-7, which was sacrificed 8 days after the secondary infection, was considered a day-8 primary-infection mouse because it showed no signs of infection after the first inoculation and had ELISA titers similar to those of day-8 infected mice. (TIF) [file ppat.1011603.s014.tif]

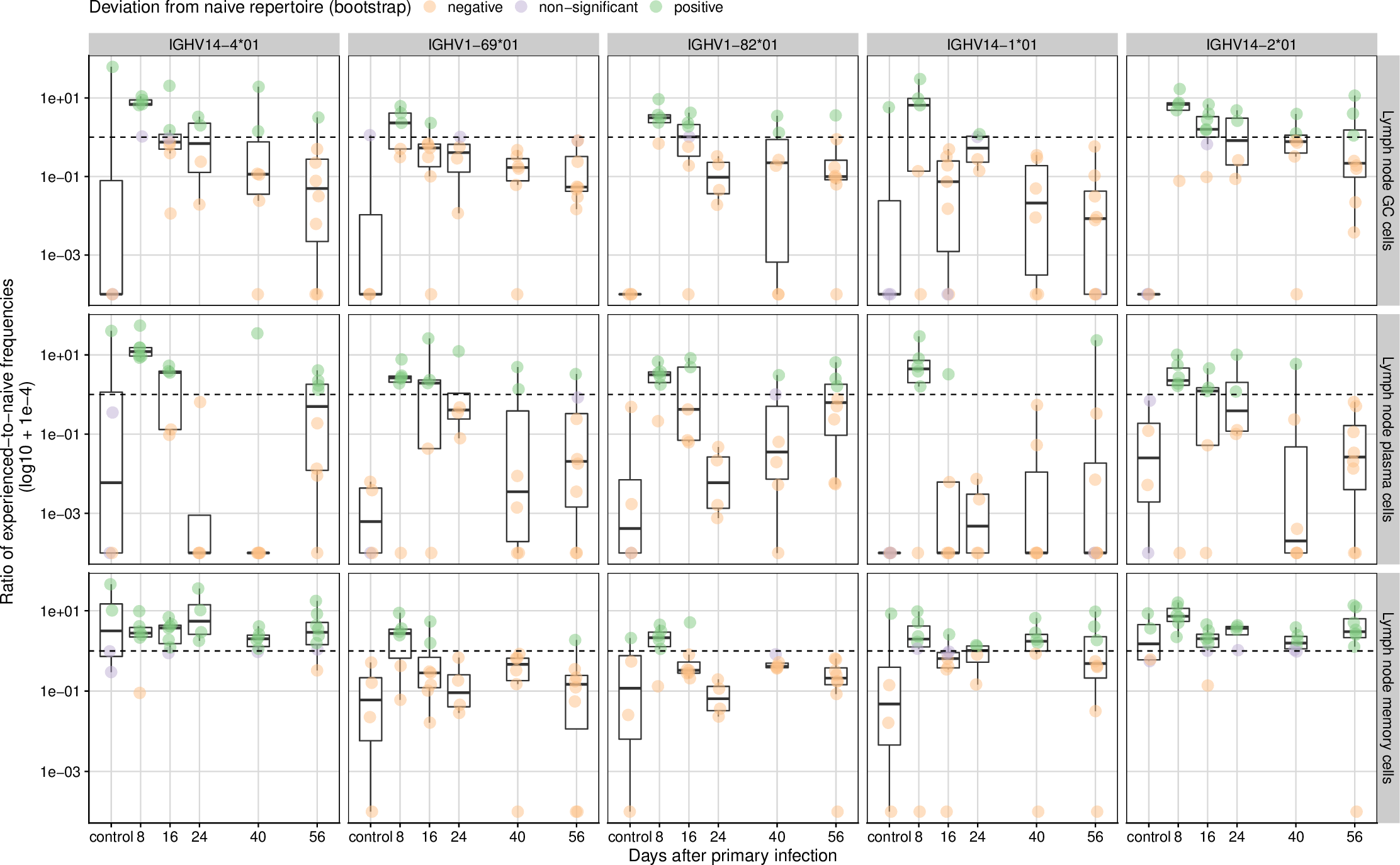

Supplement: S15 Fig — We measured frequency deviations as the ratio of the experienced-to-naive frequencies in each population. Each point represents a mouse with at least 100 sequences sampled from the corresponding experienced population and from the naive repertoire. Deviations from the naive repertoire are colored based on whether they are different from a null distribution obtained by bootstrapping experienced frequencies from the naive repertoire (n = 500 replicates) based on a 95% confidence interval test. (TIF) [file ppat.1011603.s015.tif]

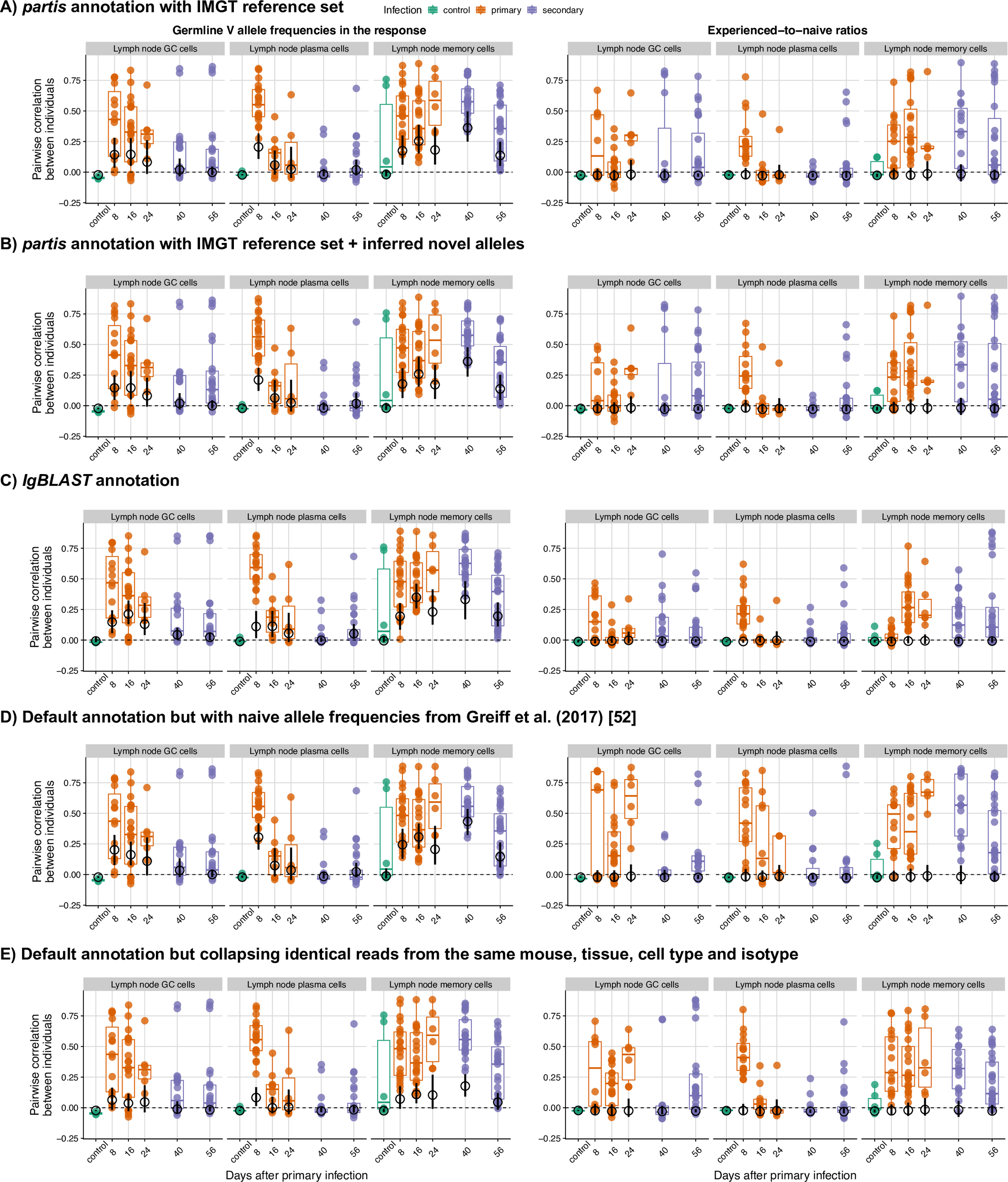

Supplement: S16 Fig — Each colored point represents a pair of mice with at least 100 reads each in the respective B cell population. The horizontal bars indicate the observed median across mouse pairs, whereas the black circles and black vertical bars indicate the bootstrap average and 95% confidence interval for the median in a null model with V alleles randomly assigned to each B cell lineage (n = 500 randomizations). We computed correlations using Pearson’s coefficient and measured frequency deviations as the ratio between a V allele’s frequency in an influenza-induced population and its frequency in the naive repertoire. (TIF) [file ppat.1011603.s016.tif]

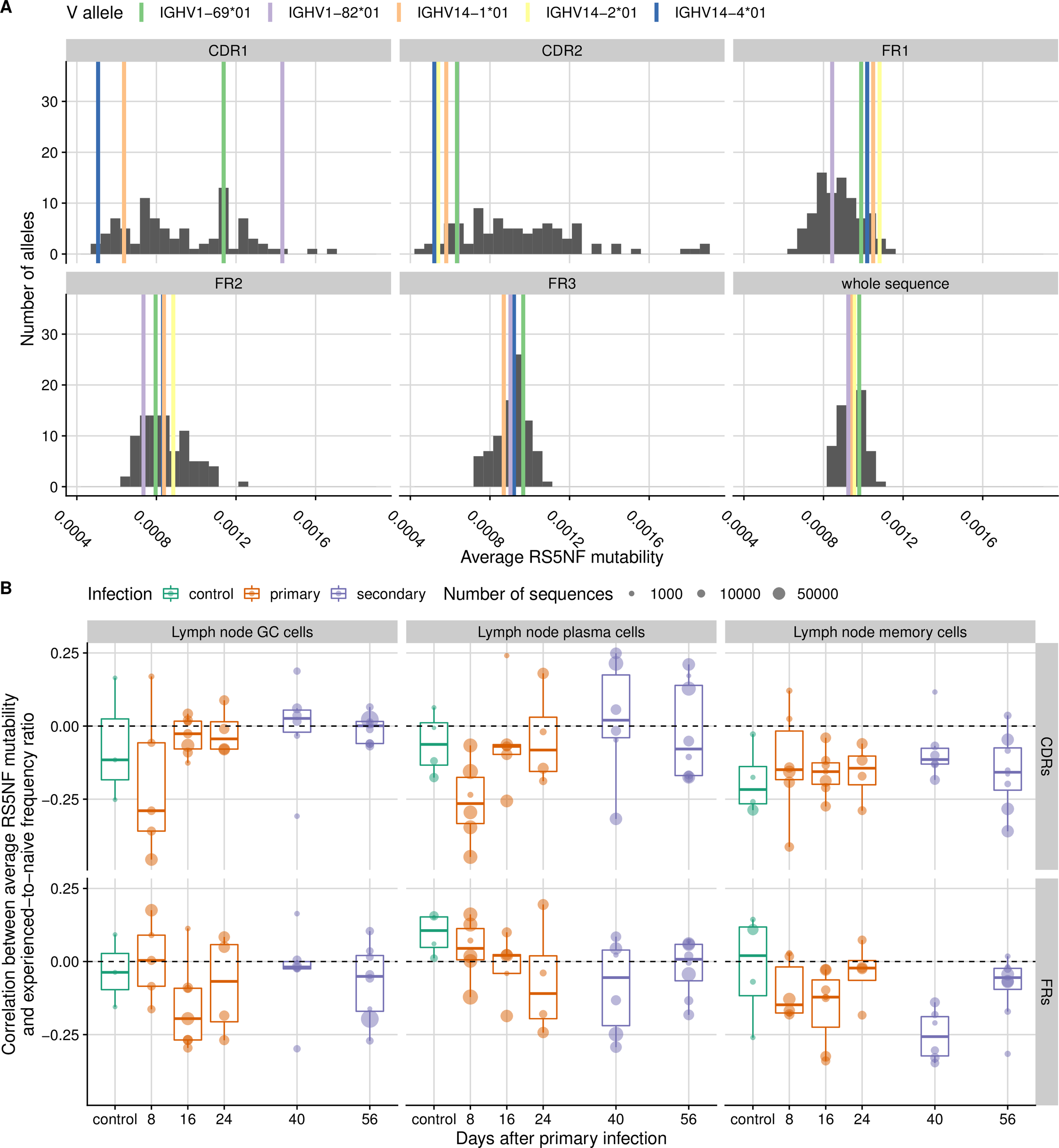

Supplement: S17 Fig — For each framework region (FR) and complementarity-determining region (CDR), we computed the average RS5NF mutability score from [54] across all 5-nucleotide motifs. In B, we computed an average across FRs weighed by the length of each FR, and similarly for CDRs. Each circle represents a mouse with at least 100 sequences each in the naive and experienced populations. Correlations were measured using Pearson’s coefficient. (TIF) [file ppat.1011603.s017.tif]

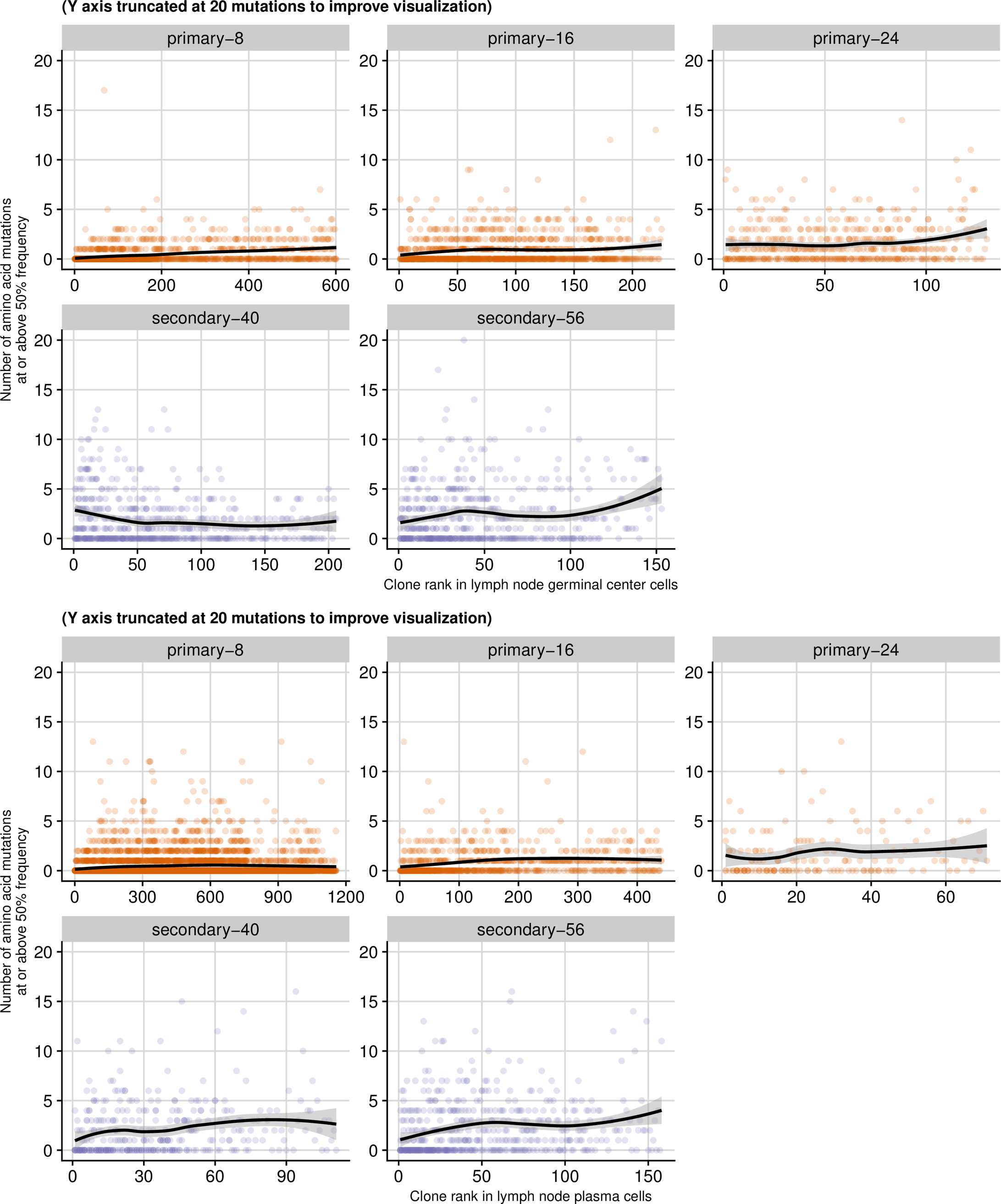

Supplement: S18 Fig — Each point represents a clone. Mice from each time point (8, 16, 24, 40 and 56 days after primary infection with influenza) were pooled together in each panel. Clone rank was determined based on the number of reads each clone had in the respective population (lymph node germinal center cells or lymph node plasma cells), not the total number of reads in the clone across all cell types and tissues (the largest clone was assigned rank 1). The solid line is a locally estimated scatterplot smoothing (LOESS) spline. (TIF) [file ppat.1011603.s018.tif]

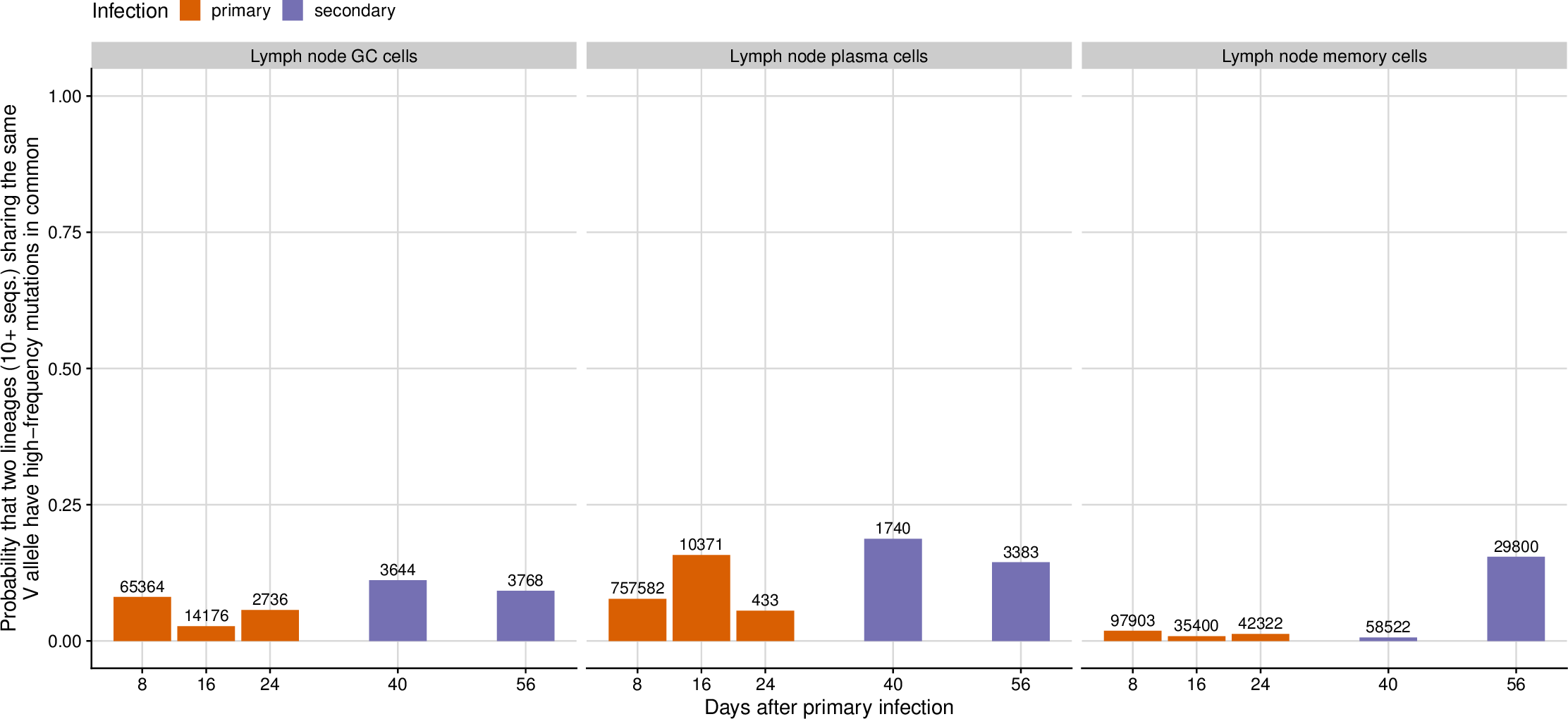

Supplement: S19 Fig — Panels represent B cell types from the lymph node of mice infected with influenza virus (GC: germinal center cells, PC: plasma cells, mem: memory cells). High-frequency mutations were those with a frequency of 50% within the lineage (considering lineage reads in each cell type). The numbers above the bars indicate the number of lineage pairs being compared (pairs were from either the same mouse or difference mice). We restricted the analysis to lineages with at least 10 reads. (TIF) [file ppat.1011603.s019.tif]

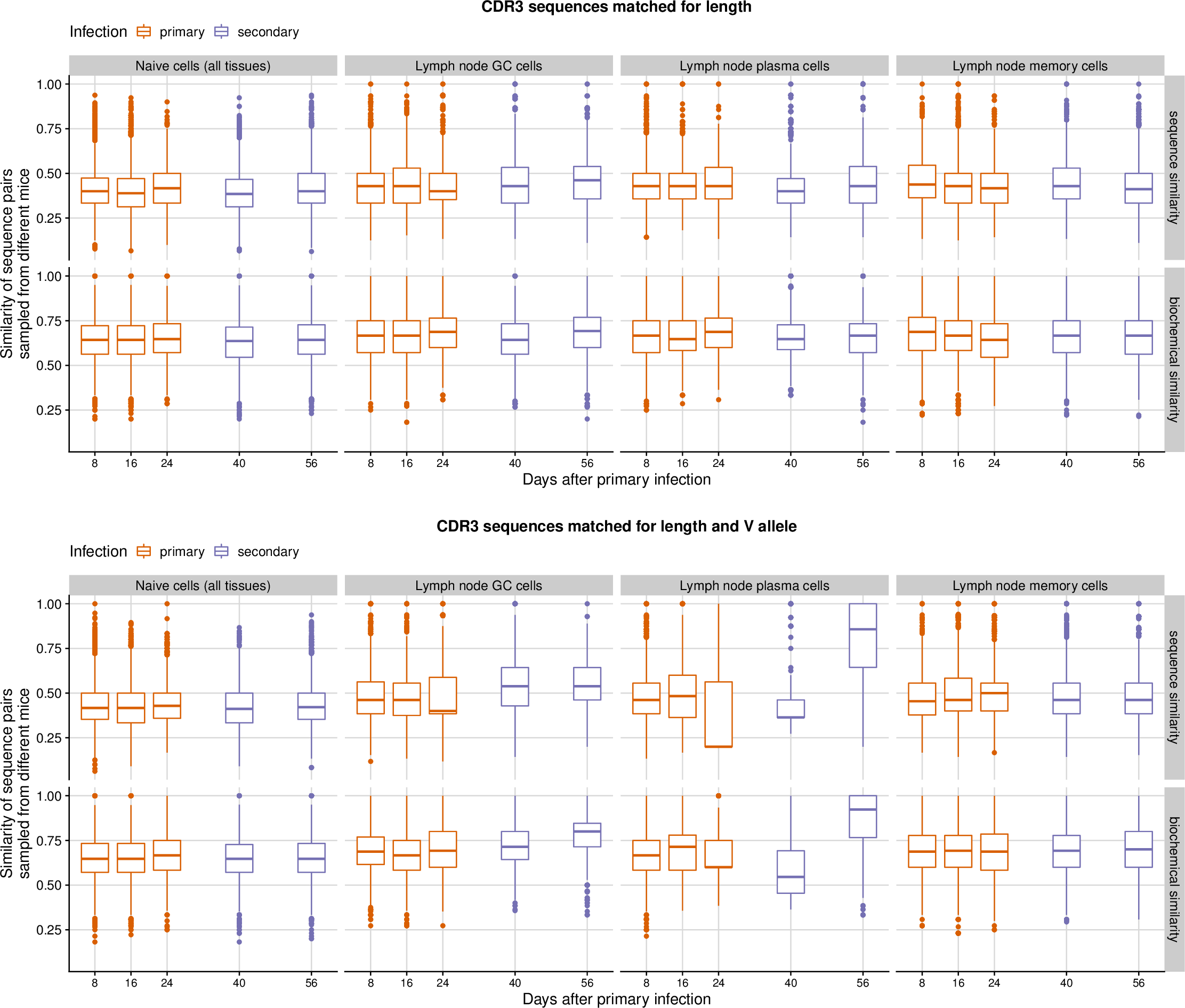

Supplement: S20 Fig — Boxplots show the distribution across sequence pairs from all mouse pairs for each time point (separately for different cell types). Values that fall outside 1.5 times the inter-quartile range are shown as individual points. (TIF) [file ppat.1011603.s020.tif]

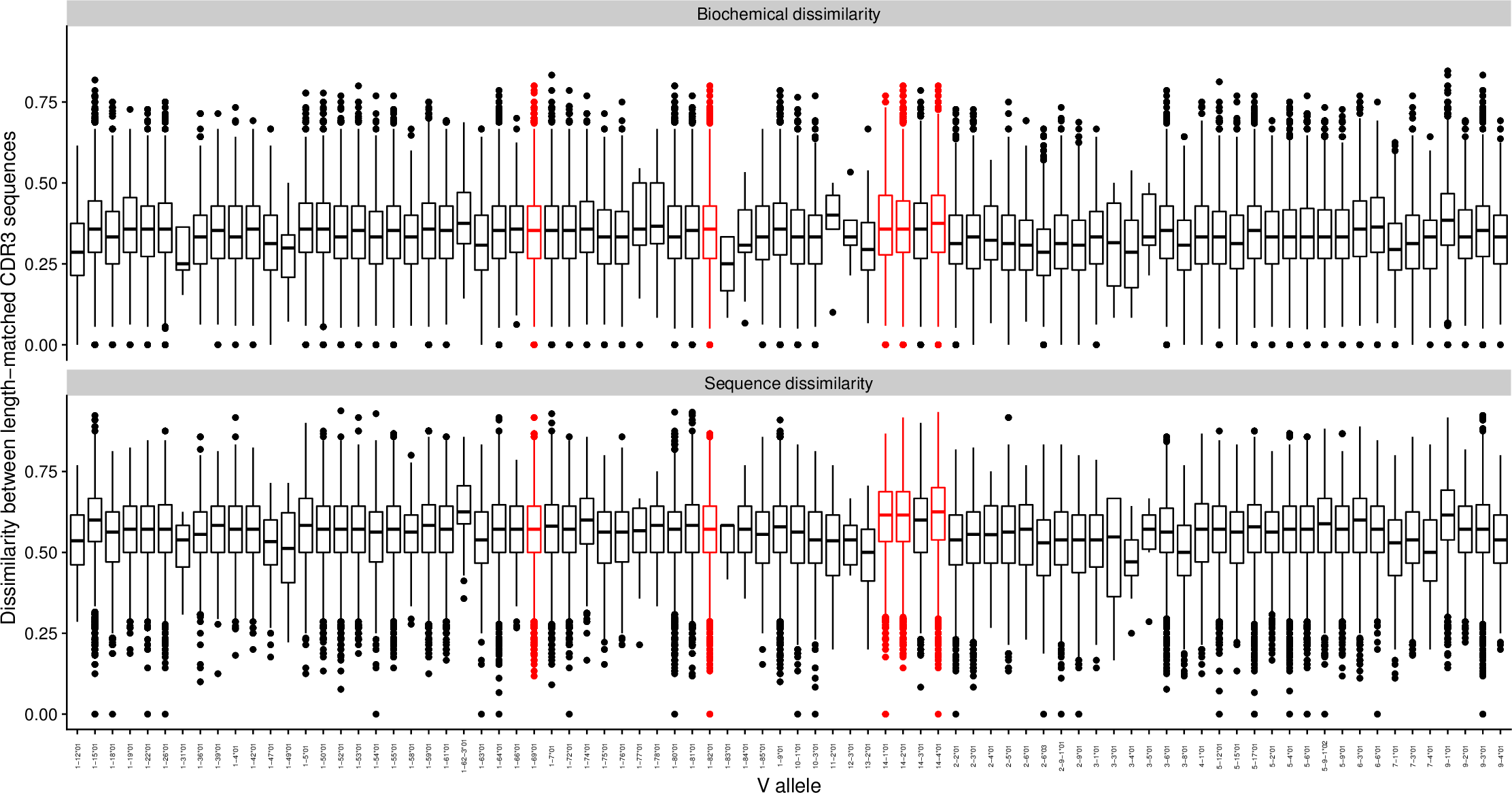

Supplement: S21 Fig — For each mouse, we identified all pairs of sequences in the naive repertoire that had the same germline allele and the same CDR3 length. For each pair, we computed the fraction of sites that had different amino acids (sequence dissimilarity, bottom), or the fraction with amino acids in different biochemical classes (top). The boxplots show the distribution of values for each V allele pooled across mice. Germline alleles consistently overrepresented in day-8 lymph node plasma cells of mouse infected with influenza are highlighted in red. (TIF) [file ppat.1011603.s021.tif]

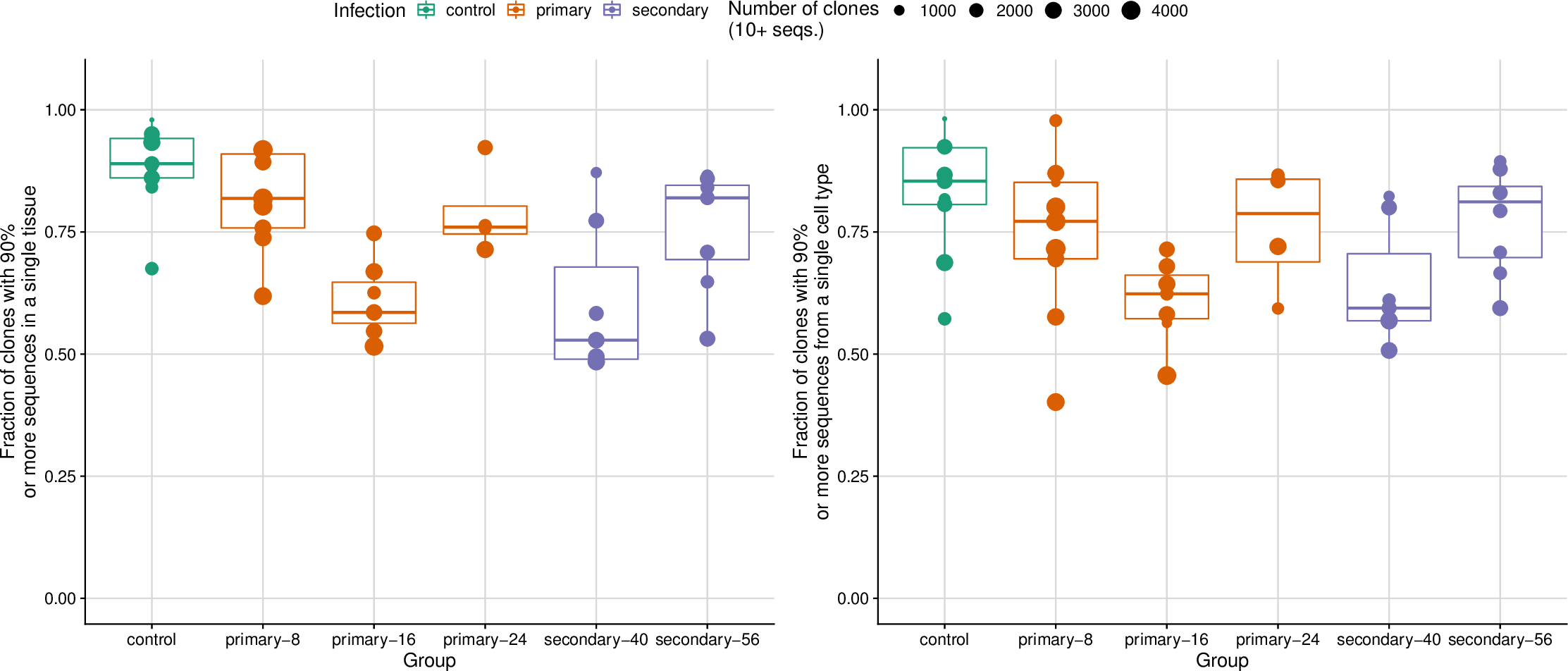

Supplement: S22 Fig — Each point represents a mouse. (TIF) [file ppat.1011603.s022.tif]

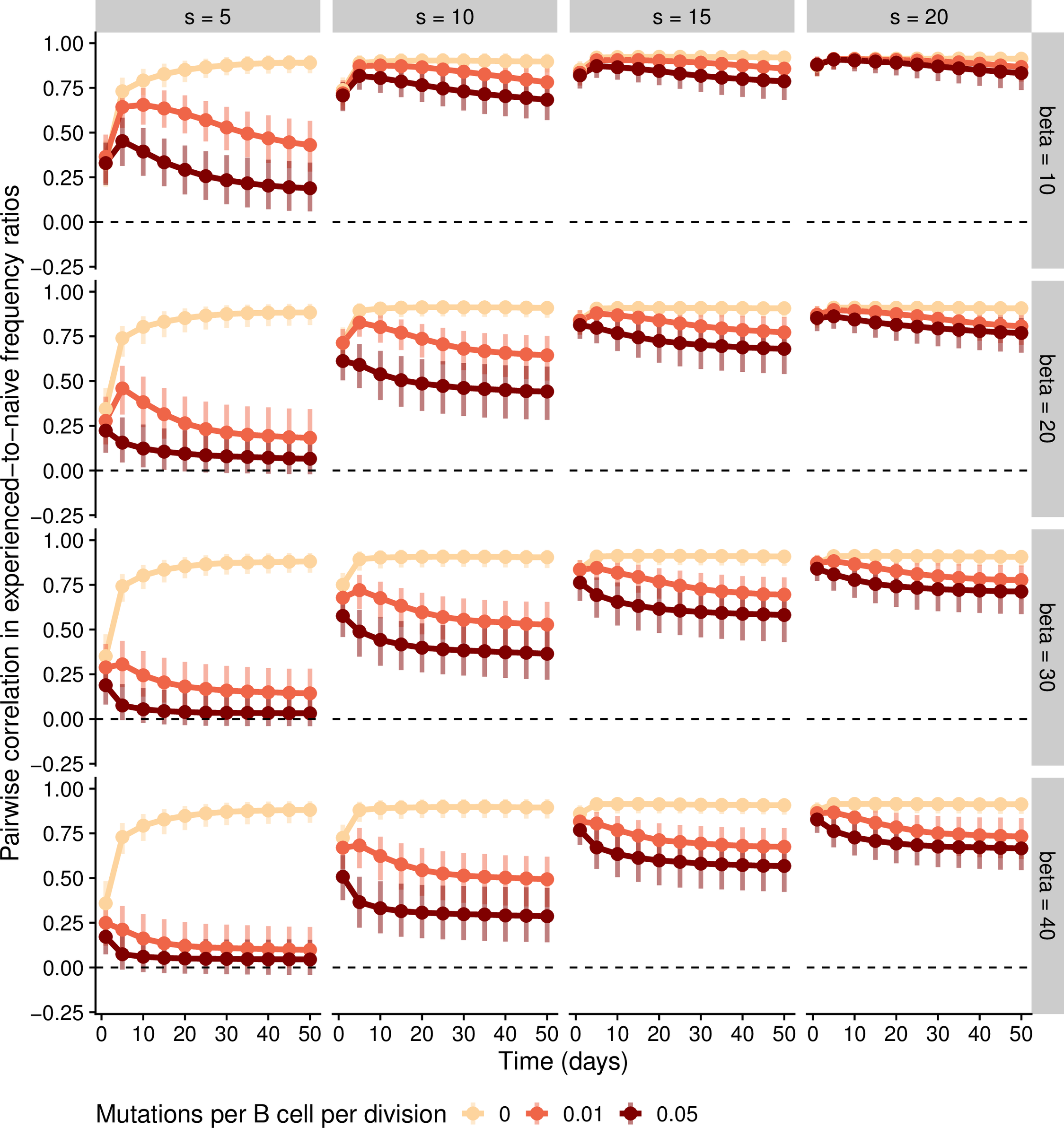

Supplement: S23 Fig — For each parameter combination, we simulated 100 individuals with varying numbers of germinal centers. We measured germline allele frequencies across germinal centers in each individual and computed the ratio between these frequencies and the allele frequencies in the naive repertoire. We then computed the correlation in these experienced-to-naive-ratios between all pairs of individuals. Points and vertical bars represent the median and the 1st and 4th quartiles of these correlations across all pairs (i.e., the bars represent true variation in simulated outcomes and not the uncertainty in the estimate of the median). For these simulations, we assumed 15 germinal centers per individual. Other parameter values are as in Table 1. (TIF) [file ppat.1011603.s023.tif]
