# Supplementary material for: Germline-encoded specificities and the predictability of the B cell response
Source: PLoS Pathog. 2023 Aug 25;19(8):e1011603. doi: 10.1371/journal.ppat.1011603 (PMC10484431; doi:10.1371/journal.ppat.1011603)
Supplement: Attachment — Submitted filename: Response_to_reviewers.pdf [file ppat.1011603.s024.pdf]

Reply to reviewers

We thank the reviewers for the thoughtful comments and suggestions. Please find detailed responses below with changes to the text highlighted in blue.

—

The report presents computational modeling and wet-lab evidence that germline encoded antibody sequences are an early determinant of the B cell response to influenza virus infection but that over time the response diversifies. The reviewers commented that the report was well-written with potentially interesting and exciting findings that may be of broad interest. Nevertheless, there was a mixed level of enthusiasm and a number of concerns were identified by each reviewer. Most notably, these include the appropriate use of statistical analyses (R1/R2,), the need to include gating strategies (R1/2), consistencies with public responses to influenza virus in humans (R1/4), accounting for the rates of SHM (R2/R3/R4), sample sizes (20) used for the modeling (R3), the validity of the conclusions and accuracy of the title (R3), and the binding specificities of the antibodies (R4). Although there are an array of issues it is likely that all of the main concerns are quite addressable and that taking these points into consideration will improve the impact and quality of the study.

Reviewer's Responses to Questions

Part I - Summary
Please use this section to discuss strengths/weaknesses of study, novelty/significance, general execution and scholarship.

Reviewer #1: Summary:

The authors measure the degree to which non-random, antibody V gene encoded responses progress overtime after infecting mice with influenza virus. Germline encoded or public antibody responses to antigen are seen in humans and can sometimes offer genetically hardwired solutions to engage conserved sites of vulnerability on hypervariable viruses including influenza. The authors apply both wetlab experiments and computational models to demonstrate that germline V gene usage dominates the early timepoints of B cell expansion in germinal centers and plasma cell responses but becomes increasingly supplanted by non-public lineages. The authors surmise that germline encoded specificities offer rapid recognition of specific antigens, but that this deterministic predictability of outcome is lower over more sustained humoral immune reactions.

Reviewer #2: The article "Increasingly divergent responses to infection in mice suggest B cell evolution is not constrained by germline-encoded specificities" by M. Viera et al. is a study investigating the constraints of Ig Variable gene usage within B cell immune responses. The authors uses modeling of B cell behavior in immune responses as a function of V gene usage to evaluate the potential roles of 'hard coded' reactivity influenced by CDRs 1 and 2 which are

germline encoded, mutability of V genes based on bioinformatic analysis of V gene sequences, and germinal center dynamics to evaluate the potential contribution of these factors to productive B cell responses. To evaluate the relative contributions of stochastic events during B cell antigen receptor diversification, the authors compare the results of these models with B cell antigen receptor sequences from the draining lymph node, spleen, and bone marrow of mice harvested longitudinally following infection with murine adapted Influenza virus.
Based on the results of this study, the authors conclude that even in situations where specific V genes are predominantly expanded early in B cell response, due to an increased frequency of antigen reactivity within certain V gene expressing receptors, continued somatic mutation and affinity maturation events result in the diversification of the antigen reactive repertoire skewing away from any Ig V gene bias.
This is a well written manuscript although there are concerns:

Reviewer #3: This is a very interesting and exciting paper that brings new evidence to bear on the question of how important germline variation is likely to be on the adaptive potential of the humoral response. The introduction and discussion are well-written. I particularly liked the pairing of simulation and experimental approaches and thought it they did indeed complement each other.. However, I am not convinced that the evidence here strongly supports the central claims of the paper. Below I discuss some critiques; for clarity, I have divided these into comments on the model and comments on the experiment.

Reviewer #4: In this manuscript, Viera and colleagues set out to study how the germline gene repertoire might constrain downstream antibody responses. They study this very important question both using simulations and experimental mouse data.

Strength: The study is extensive and I appreciate that both simulation as well as experimental data have been leveraged to investigate the research question.

Weakness: I have concerns about the simulation model used as well as the experimental data generated.

Part II – Major Issues: Key Experiments Required for Acceptance
Please use this section to detail the key new experiments or modifications of existing experiments that should be <u>*absolutely*</u> *required to validate study conclusions.*

Generally, there should be no more than 3 such required experiments or major modifications for a "Major Revision" recommendation. If more than 3 experiments are necessary to validate the study conclusions, then you are encouraged to recommend "Reject".

Reviewer #1: Overall:

This is an interesting study that attempts to wholistically and experimentally define the germline

antibody contribution to antibody response as it progresses over time. The key finding that initial germline biasing is eventually supplanted by non-public antibody responses represents an important 'dilution' principle that will be of wide interest to the field. However, critical statistically comparisons are missing and need to be included to solidify this conclusion. Moreover, the authors need to address why public antibody responses in humans appear to 'escape' this dilution effect. Essentially all of them have been identified by heavy biasing within B cell stages observed at later stages of development, post affinity maturation.

Major:

1) Figure 3C and Figure 4A, are key results indicating that V gene usage dominates early, but then becomes supplanted by non-public B cell lineages, in some cases as the primary response progresses, and also, in some cases post-secondary exposure to the virus. However, there is no statistical analyses performed to define/verify these differences and conclusions.

Response: Please note that Fig. 4A (now 5A) is the one supporting the claim of increasingly divergent allele usage between mice, measured by correlations between pairs of mice. We had performed a statistical analysis to support that claim but had shown the results in the supplement. To address the reviewer's concern, we now show those results in Fig. 5A itself, which we reproduce below. Because pairwise correlations are not statistically independent (the same mouse is represented in multiple pairs), we bootstrapped to test if these correlations were higher than expected under a null model where each B cell lineage was randomly assigned a heavy chain V allele. The point estimate and 95% bootstrap confidence interval for the median correlation are shown as black points and bars in the figure. With n = 500 bootstrap replications, the associated p-value when the observed median falls outside that bootstrap CI is <0.002.

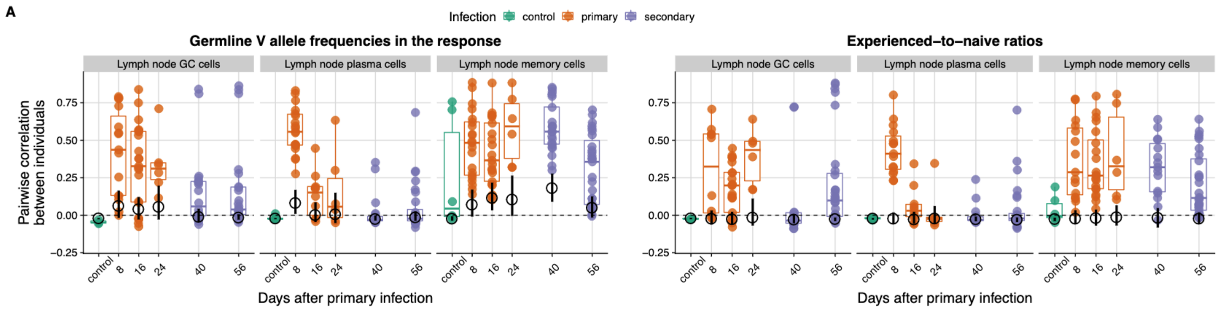

Figure 5 [Showing panel A only]. Correlation between mice in the V allele frequencies of influenza-induced populations and in the deviations of those frequencies from the naive repertoire. (A) Distribution of pairwise correlations at each time point. Each colored point represents a pair of mice with at least 100 reads each in the respective B cell population. The horizontal bars indicate the observed median across mouse pairs, whereas the black circles and black vertical bars indicate the bootstrap average and 95% confidence interval for the median in a null model with V alleles randomly assigned to each B cell lineage (n = 500 randomizations). We computed correlations using Pearson's coefficient and measured frequency deviations as the ratio between a V allele's frequency in an influenza-induced population and its frequency in the naive repertoire.

We also revised the description of those results (ll. 297-323):

“In plasma cells and germinal center cells, allele usage became increasingly dissimilar between mice over time despite evidence of germline-encoded advantages, suggesting those advantages were overcome by divergent B cell evolution in each mouse. In both cell types, the correlation in allele frequencies between mice was stronger than expected under the null model early in the response but close to the null expectation later on (Figure 5A, left panel). Early on in plasma cells, and throughout the response in germinal center cells, some germline V alleles were consistently overrepresented in different mice, leading to correlated experienced-to-naive ratios (Figure 5A, right panel; Figure S12, Figure S13) and suggesting those alleles contributed to higher affinity or adaptability than did others. For instance, in day-8 plasma cells, IGHV14-4*01 increased in frequency relative to the naive repertoire in all 6 mice with enough data, becoming the most common V allele in 3 mice and either the second or the third most common in the other 3 (Figure 5B). Later in the plasma cell response, however, experienced-to-naive ratios became uncorrelated between mice: the most common V allele was usually different in different mice, and most germline alleles were overrepresented relative to the naive repertoire in some mice but not in others (Figure S12). Taken together, these results suggest that some mouse V alleles are more likely than others to make good receptors against influenza, but that those germline-encoded advantages are not strong enough to drive persistently similar responses given the role of chance in B cell evolution.

“In contrast with plasma and germinal center cells, memory cells showed persistently biased V gene usage in influenza-infected mice (Figure 5A, Figure S14). Since activated B cells with low affinity are more likely than others to exit germinal centers and differentiate into memory cells (Viant et al., 2020), dominant lineages with high affinity for influenza antigens might contribute less to the memory cell population than they do to the germinal center and plasma cell populations. Consistent with that possibility, the increasing dominance by a few large and mutated lineages seen in germinal center and plasma cells of infected mice was not evident in their memory cells (Figure S10). Thus, memory cells at different times during the response might represent a recent sample from the naive repertoire, reflecting germline-encoded differences in initial affinity and less the divergent outcomes of affinity maturation.”

2) *Line 259. Author write “These results suggest that while germline-encoded advantages may strongly shape the early B cell response, they do not predict B cell fitness in the long run.”. This seems to be at odds with public antibody responses seen humans. Just about all of them have been discovered by the identification of over-represented V gene usage at post-affinity maturation B cell stages. How do the authors account for this if their model is to be generalized? A well described case of influenza responses includes IGHV1-69 usage, where it dominates both germline reactivity to antigen and is over represented in later stages of B cell expansion. Could it be such that different germline encoded responses dilute to different degrees?*

Response: We addressed this point by extensively revising our presentation of the simulations and experiments. Instead of referring to increasing divergence as the default expectation, we explore the conditions under which germline-encoded specificities lead to persistent versus transient overrepresentation of specific alleles. We then discuss how those conditions might differ between our study system and others. One important difference is that we study a B cell population that potentially binds multiple influenza antigens and epitopes, whereas much of the literature on public responses focuses on B cells with more restricted specificities. While these changes involved all sections of the manuscript, they are summarized in the Discussion (ll. 389-415):

“[...] In simulations, we show that whether germline-encoded advantages lead to persistently similar allele usage between individuals depends on the magnitude of those advantages relative to the effect of mutations. When mutations can easily overcome initial differences in affinity, germline allele usage diverges after an initial period of similarity. This divergence happens because B cell evolution and competition depend on factors that are largely unpredictable, such as the timing, order and effect of mutations in different lineages, genetic drift and demographic stochasticity. In mice infected with influenza, these “historical contingencies” (Gould, 1989; Blount et al., 2018) erase what seem to be relatively small germline-encoded advantages that we expect to have evolved by chance, given that influenza does not naturally infect mice [...]

“The diversity of allele usage in a B cell response likely depends on the complexity of the antigen. Although certain amino acid motifs can make germline alleles polyreactive (Hwang et al., 2014; Shiroishi et al., 2018), individual alleles are unlikely to have a consistent advantage over others across all antigens and epitopes in a pathogen (Shrock et al., 2023). The response to haptens (simple antigens with few potential epitopes) tends to be dominated by one or a few alleles (Cumano and Rajewsky, 1985, 1986), whereas the response to complex antigens can use many (Kuraoka et al., 2016). Only a few germline alleles are represented in monoclonal antibodies specific for narrowly defined sites on influenza hemagglutinin (Guthmiller et al., 2021, 2022), while tens of alleles encode monoclonal antibodies that bind epitopes in the major domains of the SARS-CoV-2 spike protein (Robbiani et al., 2020; Sakharkar et al., 2021). Many germline V alleles are used by monoclonal antibodies against the IsdB protein of Staphylococcus aureus, but antibodies targeting each epitope tend to use only one or two of them (Yeung et al., 2016).

“If responses to each epitope are consistently dominated by one or a few alleles, similarity in allele usage in the overall response might reflect the extent to which different individuals target the same epitopes [...]”

3) Figure 3C. The authors should see whether this relationship holds if the pairing comparison is performed between the different mice (expanded in mouse 1 vs expanded in mouse 2-6 etc). This will test how the truly public (donor-independent) V gene usage changes over time.

Response: We performed that comparison between mice in Fig 5A (previously 4A). We discussed the results in the response to the previous comment.

Reviewer #2:

1. The authors appear to conflate individual V gene segments and alleles throughout the manuscript. C57Bl6 mice, being an inbred strain should only express a single allele of each IGHV gene.

Response: We apologize for the confusion. We revised the Introduction to clarify our usage of the word “allele” (ll. 68-71):

“(These differences in the propensity to form high-affinity receptors may exist between germline genes occupying different loci or between different alleles occupying the same locus in different chromosomes. For simplicity, we will refer to ‘different alleles’ in both cases.)”

It is important to account for both situations because our theoretical argument also applies to differences between alleles of the same locus. For consistency, we used the same terminology when discussing the mouse experiments.

As for the germline alleles present in our mice, we switched to using the C57BL/6 germline set curated by the Open Germline Receptor Database (OGRDB) as the reference set for sequence annotation. This reference set makes no distinction between genes or alleles, since it was defined based on sequencing of expressed receptors, and therefore it is not possible to map sequences to genomic loci. Despite the expectation of identical germline sets between these inbred mice, we found variation that could not be attributed to differences in sequencing depth or to our chosen annotation tool. We revised the text to discuss that finding (ll. 264-269):

“Although C57BL/6 mice are inbred, we found differences in the set of germline V alleles between mice that could not be explained by variation in sequencing depth and that were robust to the choice of reference allele set and annotation tool (Figure S9; Methods: “Estimating the frequencies of V alleles and B cell lineages”). To our knowledge, a systematic analysis of germline variation between mice of the same inbred strain under different reference sets and annotation tools is missing, though at least one other study suggests some variation in C57BL/6 (Greiff et al. 2017).”

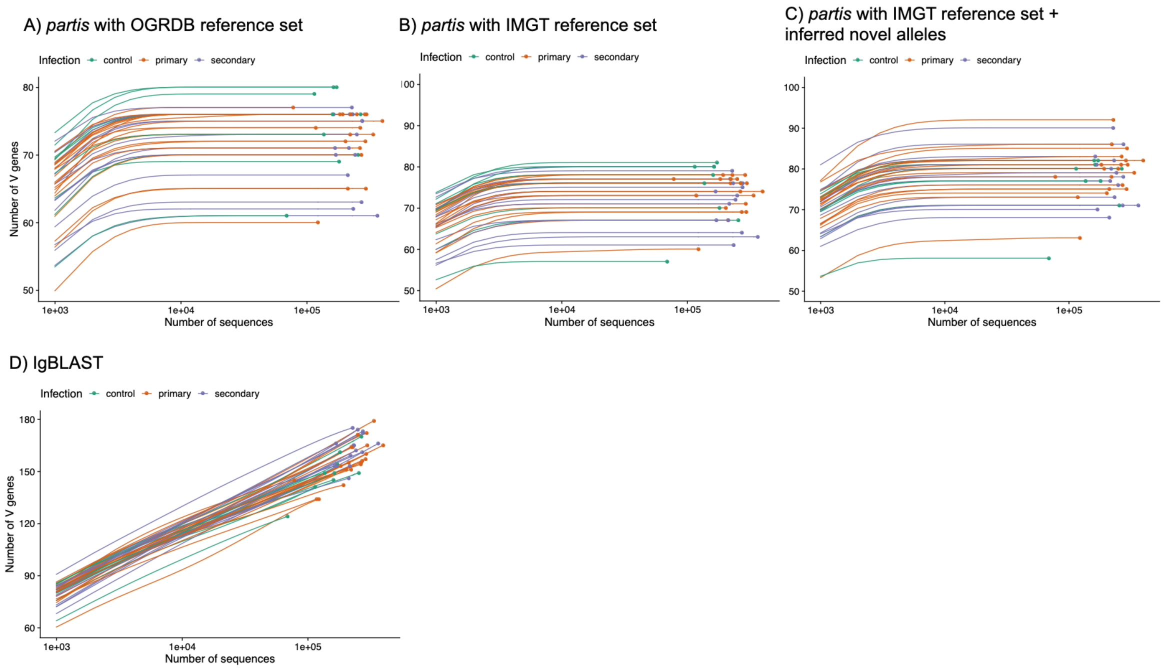

Figure S9. Number of germline V alleles detected in each mouse under different annotation tools and reference allele sets. Each circle represents the observed value for an individual mouse, with the associated rarefaction curve indicating the expected number of alleles if only a random subset of the mouse's sequences had been sampled.

2. The authors should include B cell subset sort plots from tissues in supplemental figures.

Response: We now include these plots (Fig. S7):

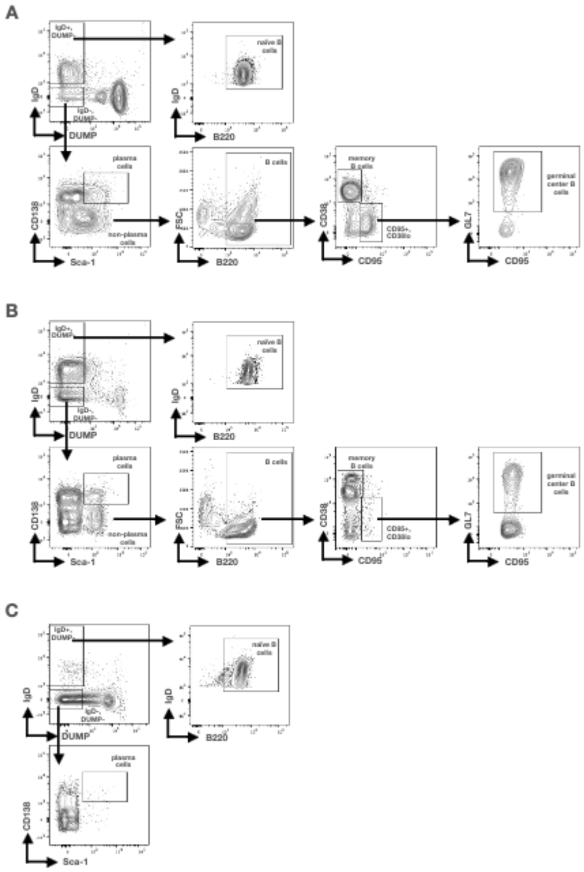

Figure S7. Representative flow cytometry plots for gating strategy for cell sorting. Representative plots for gating strategy for mediastinal lymph nodes (A), spleen (B), and bone marrow (C). Debris and doublets were first gated out (not shown), followed by exclusion of cells expressing CD4, CD8, TER-119, and/or F4/80 (DUMP). Lymph node (A) and spleen (B) IgD+DUMP- cells that were positive for B220 were sorted as naïve B cells. IgD-DUMP- cells that were Sca-1hiCD138hi were sorted as plasma cells. Sca-1lo/-CD138lo/- cells that were also B220+ were further gated and sorted as memory cells (CD95-CD38hi) or germinal center B cells (CD95+CD38lowGL7+). From bone marrow, we only sorted naïve B cells (IgD+DUMP-B220+) and plasma cells (IgD-DUMP- Sca-1hiCD138hi) (C). These representative plots show tissue from one mouse 56 days after primary infection.

3. The diversity of CDR3 sequences across V genes should be assessed. – the observation that some V genes are more rapidly recruited into influenza responses may also be explained by differences in diversity across V genes in the pre-existing repertoire.

Response: We implemented the analysis suggested by the reviewer (ll. 384-386):

“Finally, we found no evidence that the germline alleles overrepresented in the early B cell response to influenza were associated with more diverse CDR3 sequences in the naive repertoire (**Figure S21**).”

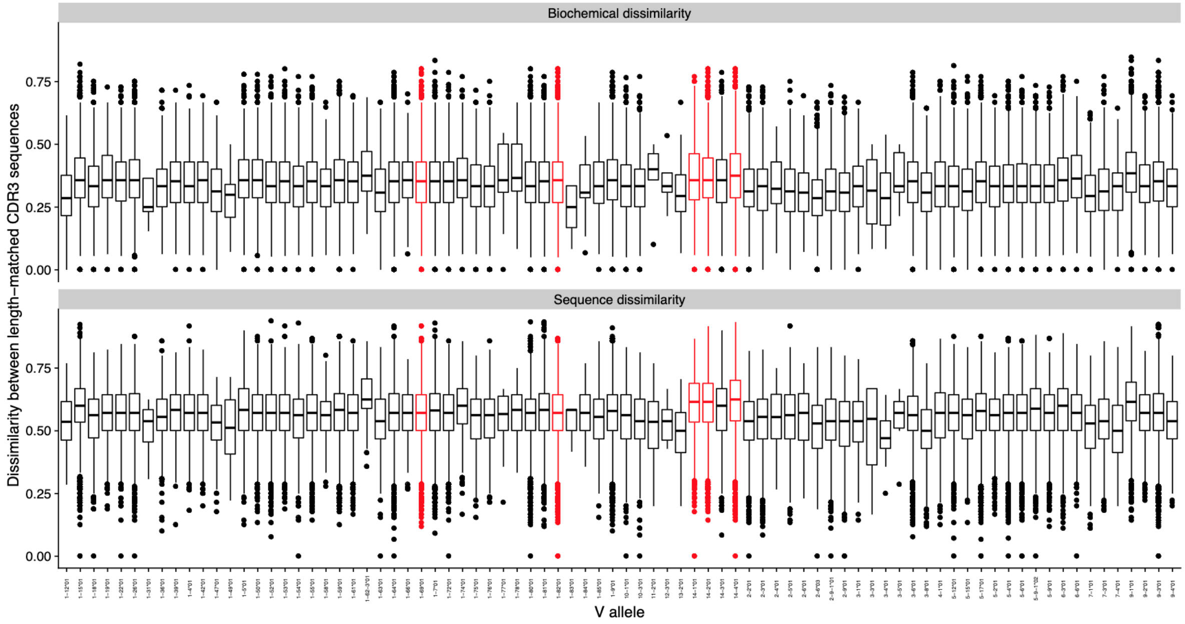

Figure S21. Diversity of CDR3 sequences in the naive repertoire for each germline heavy chain V allele. For each mouse, we identified all pairs of sequences in the naive repertoire that had the same germline allele and the same CDR3 length. For each pair, we computed the fraction of sites that had different amino acids (sequence dissimilarity, bottom), or the fraction with amino acids in different biochemical classes (top). The boxplots show the distribution of values for each V allele pooled across mice. Germline alleles consistently overrepresented in day-8 lymph node plasma cells of mouse infected with influenza are highlighted in red.

4. B cell lineage should be clearly defined (commonly defined by V and J gene identity, CDR3 length, and a percentage nucleic acid sequence similarity)

Response: We revised the text to explain how *partis* identifies B cell lineages (ll. 585-590):

“We used *partis* v0.16.0 (Ralph and Matsen 2016a,b, 2019) to partition sequences into lineages and identify the germline alleles used by each lineage’s naive ancestor. Briefly, partis identifies lineages by comparing the probability that two clusters of sequences came from a single rearrangement event with the probability that they came from separate rearrangement events. Sequences in the same lineage must have the same germline V and J segments, but *partis* does not require a minimum degree of similarity in CDR3 for sequences to be part of the same lineage.”

5. Do the rates of somatic mutation in experimental date derived from repertoire data reflect mutability scores?

Response: We don't expect the somatic mutations in the experimental data to align closely with the mutability scores, and so we have not performed this comparison. The mutability scores from Cui et al. (2016) were derived by computationally searching for mutations that could be assumed to be selectively neutral, whereas the full set of mutations in our data set includes mutations under purifying or positive selection. We used the mutability scores simply to compare germline alleles' potential mutation rates with their degree of overrepresentation in the experienced B cells of infected mice.

Reviewer #3: The model

My core critique of this model is that the stated goal (line 157) is not to make quantitative predictions based on realistic parameter values. However, the main conclusions one would draw from the model seem to depend critically on the actual values. For instance, it is clear from the simulations that if rates of SHM are low enough, then germline-based affinity matters a lot whereas if rates are high, they overwhelm any initial differences. Therefore it seems essential to know where on this spectrum we are empirically and there seems to be plenty of evidence (including in the experimental component of the paper) that could be better used to parameterize the model.

Response: Thank you for these comments. In response, we substantially revised the presentation and discussion of our simulations. Instead of describing the decrease in similarity over time as the default behavior of the model, we explore the conditions under which germline-encoded specificities lead to persistent versus transient similarity in allele usage between individuals (ll. 181-187):

"We focused on how similarity between individuals over time depends on the magnitude of germline-encoded advantages (s, defined as the increase in mean naive affinity for an allele relative to other alleles) and the rate and effect size variation of somatic mutations (with variation measured as the standard deviation β around a mean effect of zero). Different host-pathogen systems likely exist in different parts of this parameter space, since naive affinities and mutational effects are epitope-specific and depend on the host's germline alleles, their recombination and insertion/deletion probabilities, and the frequency and targeting of somatic hypermutation"

We revised all sections of the manuscript to reflect this change, but the main ideas are summarized in the last paragraph of the Introduction (ll. 100-110) and in the new Fig. 3.

"Using computational models and experiments, we investigated the role of germline-encoded specificities during the B cell response. In simulations, whether germline-encoded advantages lead to similar allele usage in different individuals depends on how large they are relative to the rate and effect size variation of somatic mutations. While large germline-encoded advantages result in persistent similarity between individuals throughout the response, affinity maturation can overcome weaker initial differences and lead to increasingly divergent responses over time

due to the stochasticity inherent to evolution. We observe this latter pattern in mice experimentally infected with influenza —a virus that does not naturally infect them— suggesting that germline-encoded specificities that arise as byproducts of evolution are initially weak and thus mostly affect the early B cell response. Selection to reinforce those specificities in the long-term evolution of jawed vertebrates might thus be driven by the fitness benefits of responding rapidly to common pathogens [...]"

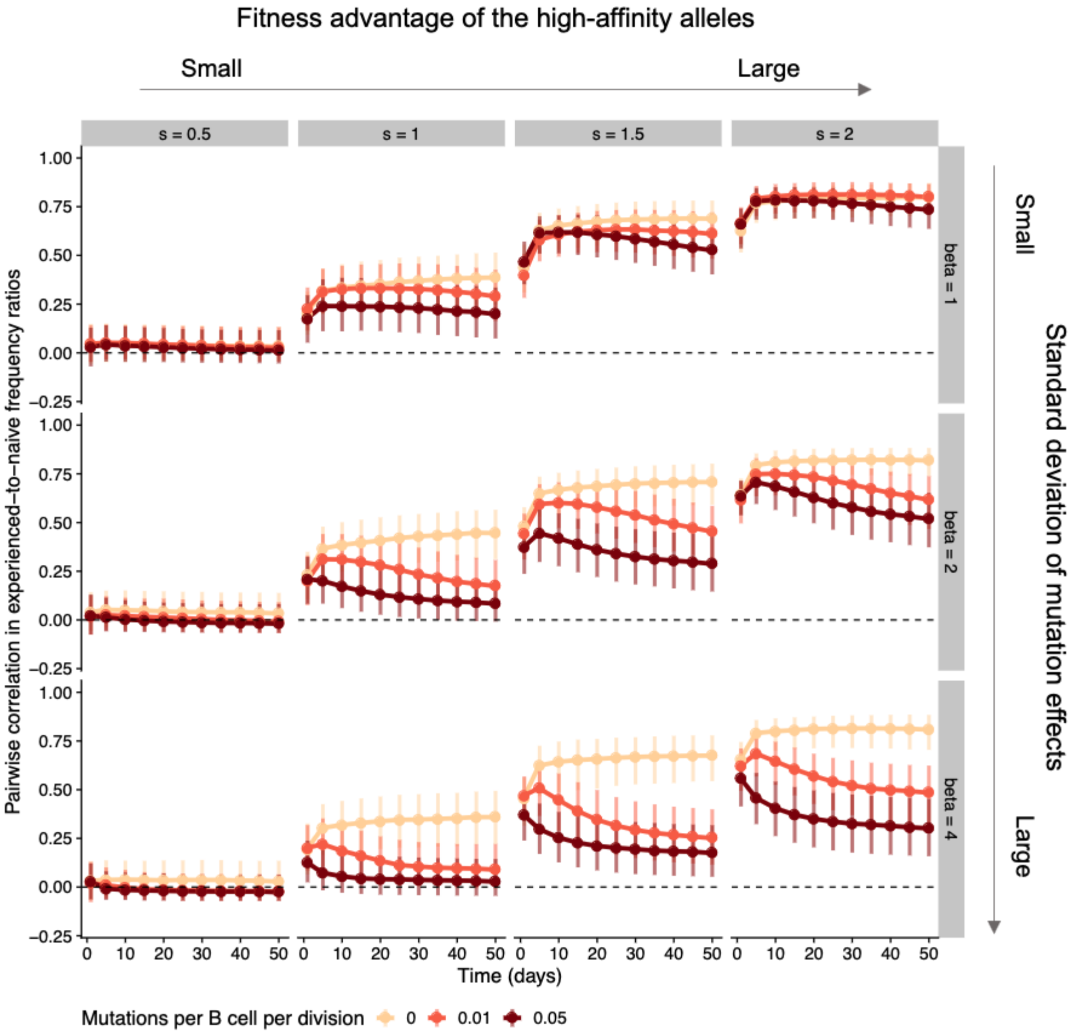

Figure 3. Simulations in the scenario where the same 5 germline alleles have higher expected affinity than the others in all individuals. For each parameter combination, we simulated 100 individuals with varying numbers of germinal centers. We measured germline allele frequencies across germinal centers in each individual and computed the ratio between these frequencies and the allele frequencies in the naive repertoire. We then computed the correlation in these experienced-to-naive-ratios between all pairs of individuals. Points and vertical bars represent the median and the 1st and 4th quartiles of these correlations across all pairs (i.e., that the bars represent true variation in simulated outcomes and not the uncertainty in the estimate of the median). For these simulations, we assumed 15 germinal centers per individual. Other parameter values are as in Table 1.

The model uses 20 individuals. Perhaps this number was chosen to match the experimental data and that may be desirable if the goal is to make quantitative predictions that are most relevant for the experiments but as above, my understanding is that the paper is investigating general theoretical properties. The beauty of simulations is that one is not constrained to realistic sample sizes so why not ramp this up considerably so that the results are less affected by statistical noise. (These simulations should be very fast if coded efficiently.) This is particularly important when the conclusion is a negative one, as in this paper.

Response: We increased the number of individuals from 20 to 100 per parameter combination. In power analyses performed on a fixed parameter combination, we found that further increasing the number to 1000 did not seem to impact the distances between the correlation curves (Fig. S1). Please note that in these curves the bars represent “real” dynamical variation in simulated outcomes (the 1st and 4th quartiles of the distribution of simulated pairwise correlations), and not the uncertainty in the estimate of the point value (the median of that distribution). While assessing this uncertainty would require computing the median for replicate runs of the same parameter combination with the same number of individuals, the stability between 100 and 1000 individuals in our power analyses suggests that this uncertainty is sufficiently small with 100 individuals. Finally, we note that the zero-mutation rate curves within a single column of Fig. 3 are effectively replicated realizations of 100 individuals (since beta does not matter when the mutation rate is zero). The similarity between those curves in each row also suggests that 100 individuals is sufficient.

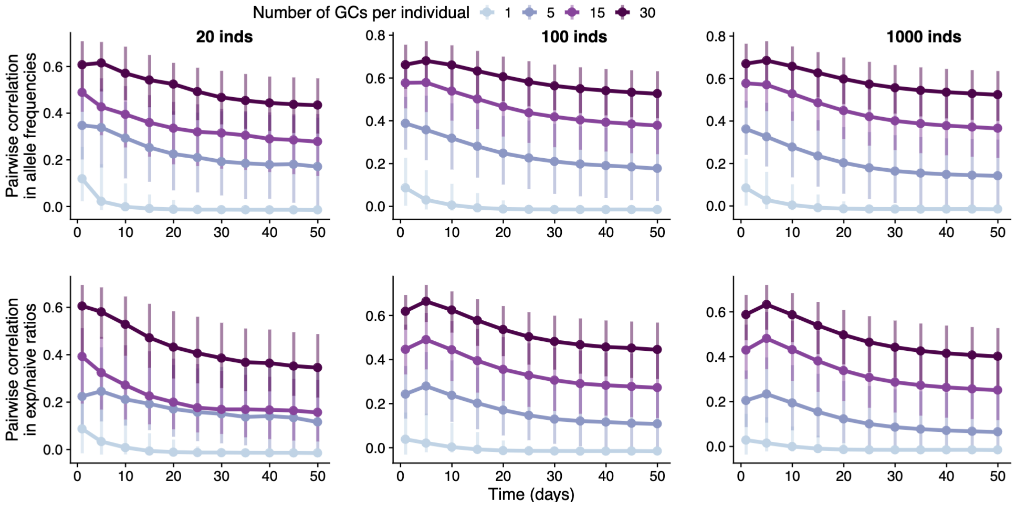

Figure S1. Simulated correlation in germline allele frequencies and experienced-to-naive ratios between different numbers of simulated individuals. We measured germline allele frequencies across germinal centers in each individual and computed the ratio between these frequencies and the allele frequencies in the naive repertoire. Points and vertical bars represent the median and the 1st and 4th quartiles of these correlations across all pairs (i.e., the bars represent true variation in simulated outcomes and not the uncertainty in the estimate of the median). We let 5 germline alleles have the mean of their naive affinity distribution increased by s = 1.5 relative to the baseline. We set the mutation rate to 0.01 mutations per B cell division and β = 4. Other parameters were set to the default values in Table 1.

In my understanding, the distributions of the naive affinity distributions of different germline alleles are identical between mice (i.e., the value of the s parameter for a given V allele was the same across replicated individuals) and that "epistasis" between a V allele and a D/J gene is represented by a distribution of affinities selected from a truncated normal distribution. Is this correct? Seems to me that there is an odd feature of the model is that when s is large, the realized variance due to epistasis is also larger as few of the samples will be cut off for being negative; the consequence would be that there is more stochasticity in the affinity binding when s is large than when s is small.

Response: Thank you for pointing out this implicit property of our model. We chose a normal distribution so we could represent high-affinity alleles by changing the mean while keeping the variance and skewness approximately constant (for distributions defined only over a non-negative domain, such as the gamma or the log-normal, changing the mean also changes the variance, the skewness, or both). However, as the reviewer points out, the variance only remains approximately constant if the original mean is far enough from zero that only a negligible fraction of values are truncated.

To test if our results were sensitive to the truncation, we repeated the simulations of the high-affinity scenario after reducing the standard deviation of the naive affinity distributions (*sigma*) from 1 to 0.1, which reduces the probability of truncation from 0.16 to effectively zero given a mean of 1. To keep the Results section concise, we put the results of this additional analysis together with its description in the Methods (ll. 514-529). Whether germline-encoded specificities change not only the mean but also other properties of the naive affinity distribution is an interesting question to be systematically addressed in the future.

"For the default values used in the analyses ($a = \sigma = 1$), the choice of a truncated normal distribution to model naive affinity distributions causes the effective variance to increase as the mean increases (since fewer values run into the truncation). To test if our results were sensitive to this effect, we repeated the simulations of the high-affinity scenario after setting $\sigma = 0.1$ (thus reducing the fraction of truncated values in the baseline distribution from 0.16 to effectively zero and causing the variance to remain approximately constant as s increases). Because the increase in mean affinity (s) and the standard deviation of mutational effects (β) are expressed as multiples of σ, we re-scaled those parameters to explore the same absolute values as in the main analysis (since σ was divided by 10, we multiplied s and β by 10).

"We found the same behavior as in the main analysis, with germline-encoded specificities leading to persistent similarity if s was sufficiently large relative to β and the mutation rate, and decreasing similarity if s was relatively small (Figure S23). Absolute increases of 0.5 and 1 in mean affinity for high-affinity alleles, which result in very low or zero correlation between individuals in the original analysis (Figure 3), now result in strong correlations. This difference is due to the fact the higher value of σ in the original simulations leads to stronger overlap between the affinity distributions of high- and low-affinity alleles."

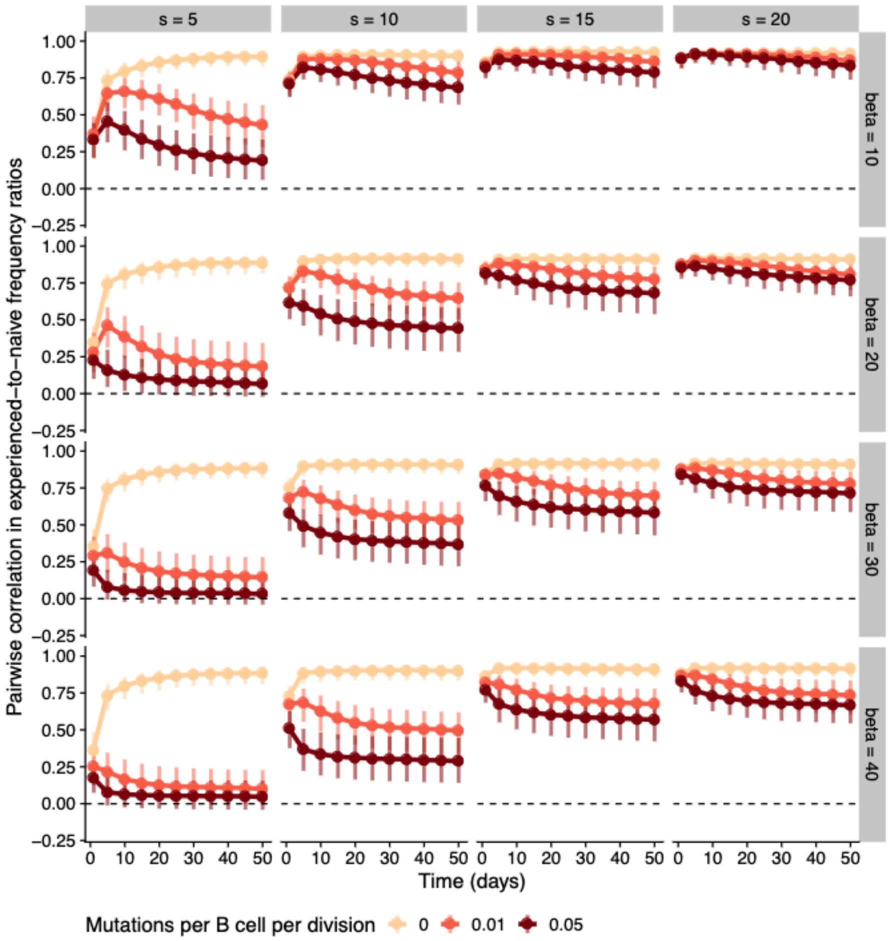

Figure S23. Simulations of the high-affinity scenario with a lower baseline standard deviation for the naive affinity distribution (σ = 0.1). For each parameter combination, we simulated 100 individuals with varying numbers of germinal centers. We measured germline allele frequencies across germinal centers in each individual and computed the ratio between these frequencies and the allele frequencies in the naive repertoire. We then computed the correlation in these experienced-to-naive-ratios between all pairs of individuals. Points and vertical bars represent the median and the 1st and 4th quartiles of these correlations across all pairs (i.e., the bars represent true variation in simulated outcomes and not the uncertainty in the estimate of the median). For these simulations, we assumed 15 germinal centers per individual. Other parameter values are as in Table 1.

I am also confused on the point as to whether the 20 individuals had identical haplotypes (as in the mouse lines used in the experimental setup) or if they shared most of them (as per line 173). A lot of the evidence for germline encoded differences in the adaptive response comes from variation between individuals of different haplotypes.

Response: Simulated individuals did not have exactly identical haplotypes. We revised the Results (ll. 201-203) to clarify that each simulated individual was based on a randomly chosen

real mouse (with replacement, since we have more simulated individuals than real ones). Since those mice had different inferred alleles sets, so did the simulated individuals.

"For each simulated individual, we randomly sampled (with replacement) a mouse for which we empirically estimated the set of heavy chain V alleles and their frequencies in the naive repertoire."

I think it is inconsistent with the terminology in the evolutionary literature to say that the "neutral" condition requires both no differences in binding affinity AND no differences in mutation rate. It seems completely consistent with a neutral scenario to have variation in the mutation rate across the genome. On this point, the germline-encoded adaptability argument laid out in the introduction suggests that different germline alleles may have different rates of beneficial and deleterious mutations owing to epistasis but in the simulations it is the pure rates of total mutations (which the paper states, line 310, have been shown not to matter) that vary among alleles and not the fitness consequences of mutations. These are conceptually distinct and in my understanding, would be described differently in a mathematical model. I think this affects our interpretation of results (e.g. line 288) does not seem well justified given the set up here.

Response: We now refer to the scenario without differences between alleles in their naive affinity distributions and mutation rates as the "functional equivalence" scenario. We agree with the reviewer that our simulations covered only one dimension of adaptability, the overall mutation rate. We revised the Results and the Discussion to acknowledge that limitation:

ll. 330-334: "Our simulations suggest that correlated experienced-to-naive ratios early in the response more likely reflect germline-encoded differences in affinity than in the overall mutation rate (Figure 3, Figure S6; we did not consider that some germline alleles may have relatively more beneficial mutations accessible to them, which might also lead to germline-encoded differences in adaptability)."

ll. 441-445: "Further understanding variation in germline gene usage requires overcoming limitations of our analyses [...] We also did not consider germline-encoded differences in adaptability other than differences in the overall mutation rate."

I am curious as to the precise effect of changes to the number of germinal centers in the model. There are two ways one might consider this: 1) it changes the effective population size of the repertoire; 2) increased competition for the germinal center increases the strength of selection. If I am understanding the model correctly, it is only through the first mechanism that changes in the number of germinal centers matter? What is the rationale behind this choice?

Response: Both mechanisms are present in our model. Because we assume no migration between germinal centers, increasing the number of GCs adds more spatial structure and therefore reduces the overall strength of selection in the repertoire (however, because the GCs

are independent, varying the number of GCs does not affect the strength of selection within each GC). We ignored migration in the interest of simplicity and computational efficiency. For the same number of germinal centers, we expect that migration would lead to stronger selection overall and stronger selection within each germinal center (with sufficiently high migration, a single lineage with the highest affinity would ultimately dominate the entire repertoire). We think the effects of migration deserve to be explored in a separate study, as acknowledged in the text (ll. 442-443):

"Migration between germinal centers (Lee et al. 2022), which we did not investigate, might also lead to faster divergence, similar to the effect of having fewer germinal centers."

The experiment

I am curious about the choice to use influenza as an experimental antigen, precisely because – as the paper states – it does not naturally infect mice. Most of the reasoning behind germline-encoded specificity is that it is an adaptive response to deal with specific pathogens threats over evolutionary time. It is not clear to me how to extrapolate from these results to cases that are primarily of interest to investigators into germline variation. I would appreciate some extended commentary in the paper as to why this choice was made and how it might impact our ability to generalize the findings.

Response: Since pathogens evolve much faster than the architecture of their hosts' immune system, we think it is important to understand germline-encoded specificities when they are exposed to less familiar antigens as well as when they might have adapted to a particular pathogen. Much of the work on public responses and germline-encoded specificities in humans comes from pathogens with which we probably have not coevolved extensively, including influenza. We argue that germline-encoded specificities against a newly encountered pathogen might be small relative to the effects of affinity maturation. Selection could then reinforce those specificities, which our simulations suggest would lead to stronger similarity in germline usage. We discuss these ideas in the revised final paragraph of the Introduction (quoted above, ll. 100-110), and in the final paragraph of the Discussion (ll. 454-468):

"Finally, if germline-encoded specificities that arise by chance are weak and thus mostly affect the early B cell response, long-term selection to reinforce them might be linked to the benefits of responding rapidly to common and/or especially harmful pathogens. Mathematical models suggest that maintaining innate defenses against a particular pathogen becomes more advantageous the more frequently the pathogen is encountered (Mayer et al., 2016). Germline alleles specific to common pathogens or pathogenic motifs might be selected, effectively hardcoding innate defenses into the adaptive immune system (Collins and Jackson, 2018). A reliable supply of receptors against common enemies might be especially important in small and short-lived organisms, which can more quickly die of infection and have fewer naive B cells with which to cover the vast space of possible pathogens (Collins and Jackson, 2018). Reinforcing germline-encoded specificities might also be especially useful when the opportunity for

adaptation is limited, as might be the case for pathogens that induce extrafollicular responses without extensive B cell evolution (although affinity maturation can occur outside of germinal centers; Di Niro et al. 2015; Elsner and Shlomchik 2020). Understanding what conditions favor similar versus contingent allele usage in the antibody repertoire may thus shed light on the long-term evolution of immunoglobulin genes."

I do not understand the conclusions of the comparison of the estimated naive V allele frequencies sampled by Greiff et al. 2017? I would like to see an expanded discussion of this in the text; my reasoning is that I am somewhat worried that the correlations between V allele frequencies among individuals that are observed are lower than what we would expect from inbred mouse lines based on Greiff's study as well as others.

Response: We revised the text to clarify the purpose of this analysis (ll. 264-273):

"Although C57BL/6 mice are inbred, we found differences in the set of germline V alleles between mice that could not be explained by variation in sequencing depth and that were robust to the choice of reference allele set and annotation tool (Figure S9; Methods: "Estimating the frequencies of V alleles and B cell lineages"). To our knowledge, a systematic analysis of germline variation between mice of the same inbred strain under different reference sets and annotation tools is missing, though at least one other study suggests some variation in C57BL/6 (Greiff et al. 2017). Despite the variation we observed, mice had similar sets of germline alleles present at correlated frequencies in the naive repertoire (Figure 4). Because this correlation was nonetheless weaker than previously reported by Greiff et al. (2017), we repeated the analysis using data from that study to compute germline allele frequencies in the naive repertoire."

The results are now described in ll. 324-327 and shown in Fig. S16D along with other sensitivity analyses:

"We found similar results using a different germline annotation tool and different germline reference sets, using independent sequence data from Greiff et al. (2017) to estimate naive allele frequencies, and when computing allele frequencies based only on unique sequence reads (collapsing identical reads from the same mouse, tissue, cell type and isotype) (**Figure S16**)."

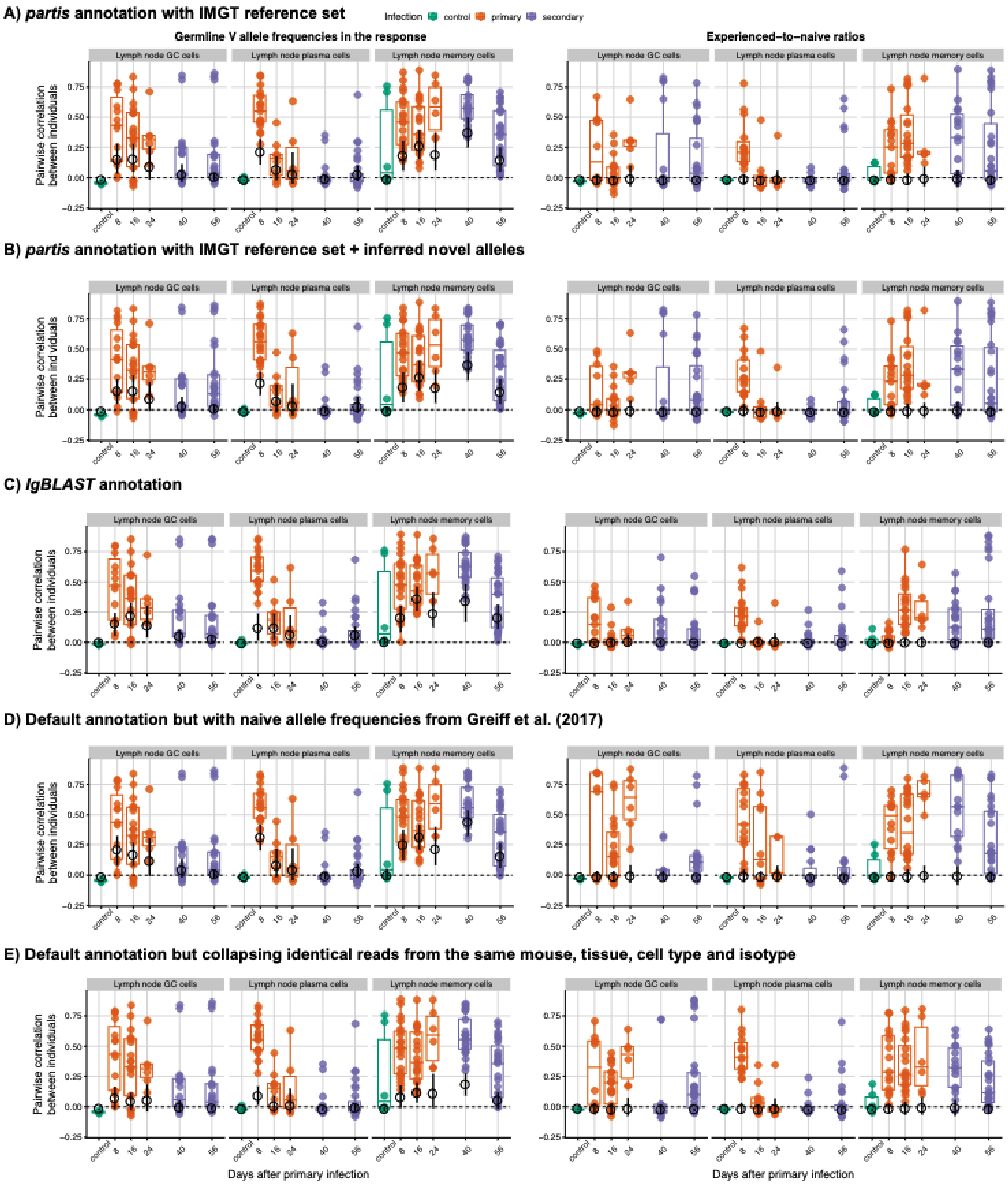

Figure S16. Correlations between mice in germline V allele frequencies and their deviations from the naive repertoire in sensitivity analyses show robustness of results to germline annotation tool and allele reference set. Each colored point represents a pair of mice with at least 100 reads each in the respective B cell population. The horizontal bars indicate the observed median across mouse pairs, whereas the black circles and black vertical bars indicate the bootstrap average and 95% confidence interval for the median in a null model with V alleles randomly assigned to each B cell lineage (n = 500 randomizations). We computed correlations using Pearson’s coefficient and measured frequency deviations as the ratio between a V allele’s frequency in an influenza-induced population and its frequency in the naive repertoire.

Reviewer #4: Experimental data:

- Can you comment on to what extent you can study your question in an experimental system where influenza is not a common pathogen? Could your conclusions maybe have been different in human where there exists a lot of germline gene variability for IGHV1-69, for example?

Response: Since pathogens evolve much faster than the architecture of their hosts' immune system, we think it is important to understand germline-encoded specificities when they are exposed to less familiar antigens as well as when they might have adapted to a particular pathogen. Much of the work on public responses and germline-encoded specificities in humans comes from pathogens with which we probably have not coevolved extensively, including influenza. We argue that germline-encoded specificities against a newly encountered pathogen might be small relative to the effects of affinity maturation. Selection could then reinforce those specificities, which our simulations suggest would lead to stronger similarity in germline usage. We discuss these ideas in the revised final paragraph of the Introduction (ll. 100-110),

"Using computational models and experiments, we investigated the role of germline-encoded specificities during the B cell response. In simulations, whether germline-encoded advantages lead to similar allele usage in different individuals depends on how large they are relative to the rate and effect size variation of somatic mutations. While large germline-encoded advantages result in persistent similarity between individuals throughout the response, affinity maturation can overcome weaker initial differences and lead to increasingly divergent responses over time due to the stochasticity inherent to evolution. We observe this latter pattern in mice experimentally infected with influenza —a virus that does not naturally infect them— suggesting that germline-encoded specificities that arise as byproducts of evolution are initially weak and thus mostly affect the early B cell response. Selection to reinforce those specificities in the long-term evolution of jawed vertebrates might thus be driven by the fitness benefits of responding rapidly to common pathogens [...]"

and in the final paragraph of the Discussion (ll. 454-468):

"Finally, if germline-encoded specificities that arise by chance are weak and thus mostly affect the early B cell response, long-term selection to reinforce them might be linked to the benefits of responding rapidly to common and/or especially harmful pathogens. Mathematical models suggest that maintaining innate defenses against a particular pathogen becomes more advantageous the more frequently the pathogen is encountered (Mayer et al., 2016). Germline alleles specific to common pathogens or pathogenic motifs might be selected, effectively hardcoding innate defenses into the adaptive immune system (Collins and Jackson, 2018). A reliable supply of receptors against common enemies might be especially important in small and short-lived organisms, which can more quickly die of infection and have fewer naive B cells with which to cover the vast space of possible pathogens (Collins and Jackson, 2018). Reinforcing germline-encoded specificities might also be especially useful when the opportunity for

adaptation is limited, as might be the case for pathogens that induce extrafollicular responses without extensive B cell evolution (although affinity maturation can occur outside of germinal centers; Di Niro et al. 2015; Elsner and Shlomchik 2020). Understanding what conditions favor similar versus contingent allele usage in the antibody repertoire may thus shed light on the long-term evolution of immunoglobulin genes."

We agree with the reviewer that the results might be different in populations with more germline gene variability, in which case similarity between individuals might decay even faster when germline-encoded advantages are relatively weak.

- Fig 3b (right plot): why is there so much germline gene usage variation? I would have expected germline gene usage to be more reproducible across mice.

Response: We revised the results to address this point (ll. 264-273):

"Although C57BL/6 mice are inbred, we found differences in the set of germline V alleles between mice that could not be explained by variation in sequencing depth and that were robust to the choice of reference allele set and annotation tool (Figure S9; Methods: "Estimating the frequencies of V alleles and B cell lineages"). To our knowledge, a systematic analysis of germline variation between mice of the same inbred strain under different reference sets and annotation tools is missing, though at least one other study suggests some variation in C57BL/6 (Greiff et al. 2017)."

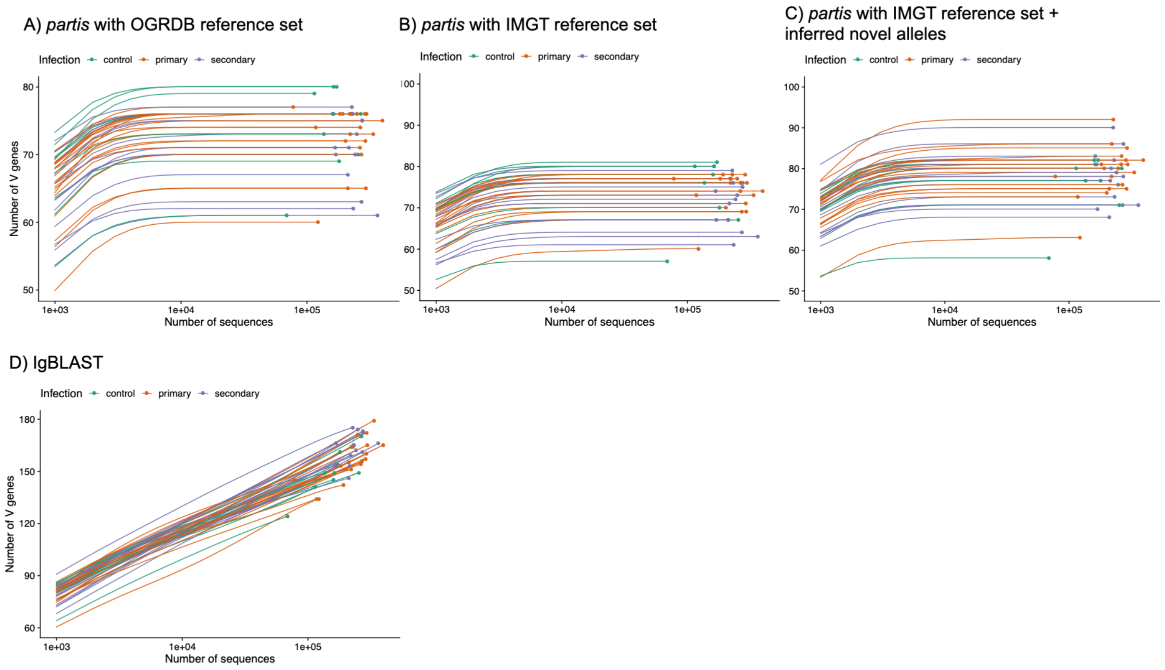

Figure S9. Number of germline V alleles detected in each mouse under different annotation tools and reference allele sets. Each circle represents the observed value for an individual mouse, with the

associated rarefaction curve indicating the expected number of alleles if only a random subset of the mouse’s sequences had been sampled.

- You state that "Influenza antigens do not strongly select for specific CDR3 sequences" --> but the hallmark of antibody sequence is that they can look sequence-dissimilar but are structurally similar? did you test for that?

Response: We focused on sequence similarity because we are interested in how many paths in sequence space lead to the same general phenotype (binding an antigen well, measured by HAI titers). The fact that CDR3 sequences do not seem to converge suggests there are many such paths, which we find interesting even if, as the reviewer points out, those paths might be very similar in terms of structure. With sequence data alone, it is of course hard to say much about structure. We did look at a rough measure of biochemical similarity, using a classification of amino acids into hydrophobic, hydrophilic and neutral sets, as explained in the Methods (ll. 665-668). Fig. S20 shows that CDR3s of experienced lymph node populations of infected mice do not appear more similar under that metric than the CDR3s of naive B cells.

“Following previous work (Hershberg and Shlomchik, 2006; Saini and Hershberg, 2015), we measured biochemical similarity as the proportion of sites in which the amino acids of both sequences belonged to the same category in the classification by (Chothia et al.,1998): hydrophobic (F, L, I , M, V, C, W), hydrophilic (Q, R, N, K, D, E) or neutral (S, P, T, A, Y, H, G).”

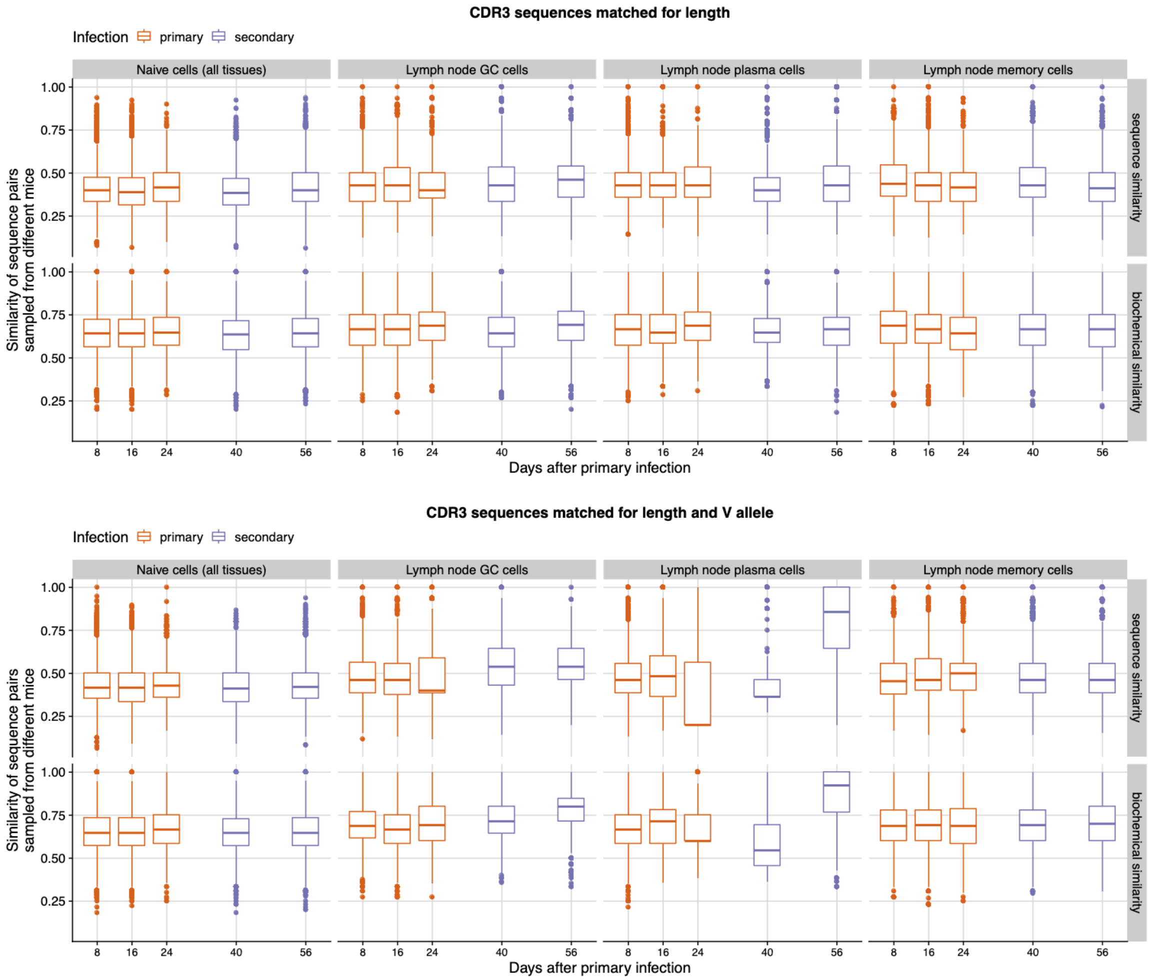

Figure S20. Similarity of CDR3 sequence pairs sampled from different mice and matched for the same length (top) or the same length and the same V allele (bottom). Boxplots show the distribution across sequence pairs from all mouse pairs for each time point (separately for different cell types). Values that fall outside 1.5 times the interquartile range are shown as individual points

- Since you don't know the specificity of the antibody sequences studied, it's very difficult to draw strong conclusions from the data. Can you comment on that? In other words, how can you study constriction of antibody repertoire dynamics without knowing the binding landscape (especially given the complex sequence-structure-binding map of antibodies)?

Response: We agree with the reviewer that not knowing the specificity or affinity of antibody sequences for particular epitopes is a limitation of our experiment, which we acknowledge in the Discussion (ll. 448-453):

"Additional experiments could estimate the epitope-level specificity and affinity distribution of naive B cells using different germline alleles, compare variation within and between those distributions, and directly test if alleles with higher affinity distributions tend to be used by B cell

lineages with high growth rates. Fine-scale specificity and affinity measurements could also help determine how much divergence in allele usage derives from differences in epitope targeting and whether certain germline alleles maintain persistent advantages with each epitope."

We also revised the Discussion (ll. 413-415) to acknowledge that the similarity in allele usage might reflect the extent to which the same epitopes are immunodominant in different individuals, which we cannot know from sequence data alone:

"If responses to each epitope are consistently dominated by one or a few alleles, similarity in allele usage in the overall response might reflect the extent to which different individuals target the same epitopes [...]"

Finally, while we cannot guarantee we have only looked at influenza-specific sequences, we have looked at populations likely to be highly enriched for them given comparisons to controls (ll. 256-260):

"While we did not sort cells based on the ability to bind influenza, we found that uninfected controls had very few germinal center, plasma or memory cells in the mediastinal lymph node (Figure S7, Figure S8), suggesting the lymph-node B cells of infected mice were induced by influenza in agreement with previous work (Sealy et al., 2003). We therefore focused on lymph node B cells, with potentially many different specificities for influenza antigens."

Simulation model

- did you account for the fact that VDJ recombination generates sequences according to a distribution (pgen, generation probability).

Response: Yes, we represented this variation implicitly by having naive B cells with the same germline V gene have a distribution of possible affinities:

"To represent variation in germline-encoded affinity, B cells using different heavy chain germline V alleles can have different naive affinity distributions. The variation within each distribution in turn represents the effects of stochasticity in VDJ recombination and the pairing of heavy and light chains."

- SHM: did you account for the fact that SHM has hotspot motifs and even germline-gene-specific motifs?

Response: Our simulations did not explicitly model antibody sequences, only affinity as a phenotype, and therefore we did not represent hotspot motifs explicitly. However, we considered a scenario where BCRs using specific germline alleles had higher overall mutation rates than others. In the real world, one potential mechanism behind these differences would be differences in the number and distribution of hotspot motifs across germline alleles.

Part III – Minor Issues: Editorial and Data Presentation Modifications
Please use this section for editorial suggestions as well as relatively minor modifications of existing data that would enhance clarity.

Reviewer #1:

1) The authors mention the role stochasticity can play. Does the authors model also account for the resultant permissiveness in B cell selection? The monitoring of clonal composition within a primary GC reveals that 'winner-take-all' events are rare, and that stochastic factors unrelated to BCR affinity can strongly influence selection, enabling non-homogenizing B cell selection and longer term survival of low affinity B cell clones (PMID 26912368).

Response: Yes, the stochasticity in our simulations leads to variation between germinal center in how strongly they are dominated by a single "winner" lineage. Although not a focus of our exploration, this variation is shown in the vertical bars of Fig. 2A:

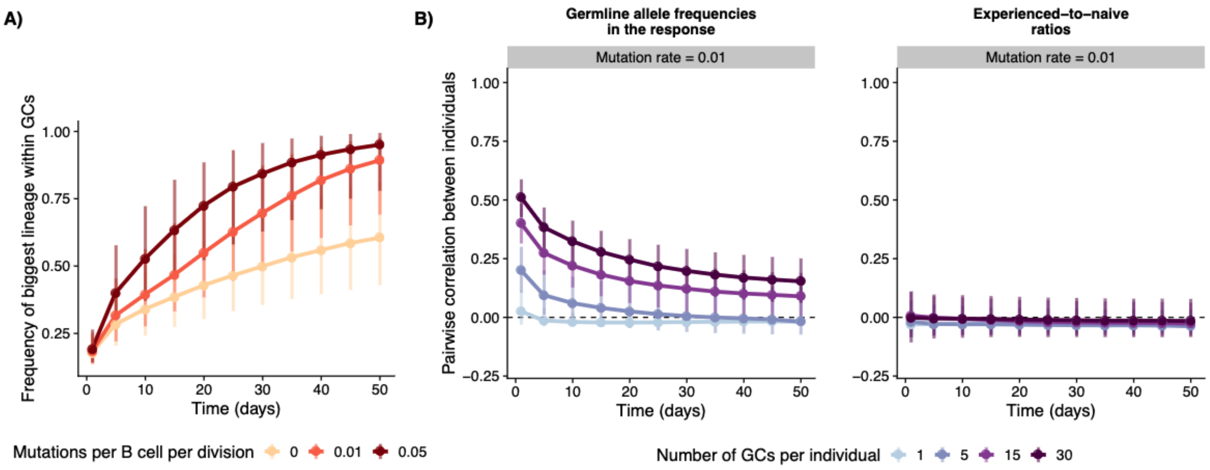

Figure 2. Simulations in the scenario with identical naive affinity distributions and mutation rates across germline alleles. For each parameter combination, we simulated 100 individuals with varying numbers of germinal centers. A) For each germinal center, we computed the fraction of the total germinal center population occupied by the biggest lineage. Points and vertical bars represent the median and the 1st and 4th quartiles for this fraction across simulated germinal centers. B) We measured germline allele frequencies across germinal centers in each individual and computed the ratio between these frequencies and the allele frequencies in the naive repertoire. We then computed the correlation in frequencies and experienced-to-naive-ratios between all pairs of individuals. Points and vertical bars represent the median and the 1st and 4th quartiles of these correlations across all pairs (note that the bars represent true variation in simulated outcomes and not the uncertainty in the estimate of the median). We set β = 2 and left other parameters as shown in Table 1

2) The authors should include representative flow plots for sorting of the different B cell: the naïve B cells and the GC, memory, and plasma cells that were expanded post-infection.

Response: We have included these plots (Figure S7):

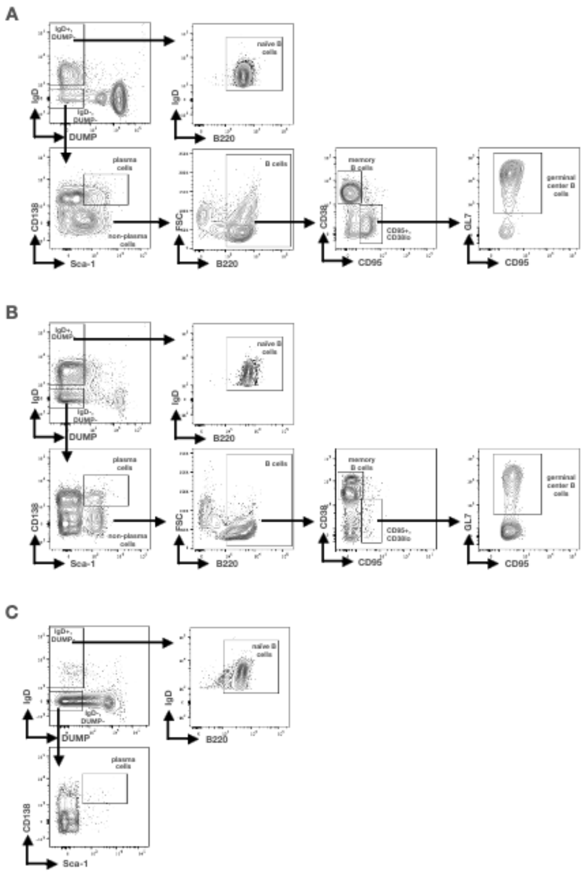

Figure S7. Representative flow cytometry plots for gating strategy for cell sorting. Representative plots for gating strategy for mediastinal lymph nodes (A), spleen (B), and bone marrow (C). Debris and doublets were first gated out (not shown), followed by exclusion of cells expressing CD4, CD8, TER-119, and/or F4/80 (DUMP). Lymph node (A) and spleen (B) IgD+DUMP- cells that were positive for B220 were sorted as naïve B cells. IgD-DUMP- cells that were Sca-1hiCD138hi were sorted as plasma cells. Sca-1lo/-CD138lo/- cells that were also B220+ were further gated and sorted as memory cells (CD95-CD38hi) or germinal center B cells (CD95+CD38lowGL7+). From bone marrow, we only sorted naïve B cells (IgD+DUMP-B220+) and plasma cells (IgD-DUMP- Sca-1hiCD138hi) (C). These representative plots show tissue from one mouse 56 days after primary infection.

3) Line 233 (as an example). The authors seem to equate somatic hypermutation (SHM) with affinity maturation. But these are two distinct features. SHM is a repertoire-diversifying process that provides substrate for affinity maturation, but it can easily lead to less fit/lower affinity clones.

Response: We agree that mutations can disrupt affinity, but in that case they are unlikely to reach high frequencies within a lineage. By looking at mutations present in at least half of a lineage's sequences, we attempt to look only at beneficial or neutral mutations. Without affinity maturation, we would not expect the number of these high-frequency mutations to increase over time. The fact that this increase happens in the mediastinal lymph node but not in other tissues (Fig. S11) further suggests at least some of those high-frequency mutations increase affinity against influenza antigens. We revised ll. 354-356 to indicate we are looking at high-frequency mutations and not all mutations:

"While germinal center cells and plasma cells were increasingly dominated by large lineages with somatic mutations, the sheer number of high-frequency mutations acquired by a B cell lineage did not predict its success."

4) Line 315 "Most pairs of lineages with the same V allele had no high-frequency mutations in common". This again seems at odds with some human public antibody responses, which do often contain public mutations that confer gain of function.

Response: Our results do not conflict with those previous observations. We revised the text to explain why (ll. 365-369):

"Independent acquisition of the same mutation has been shown in lineages using the same heavy chain V allele and recognizing the same antigen or epitope (Krause et al., 2011; Jackson et al., 2014; Pappas et al., 2014). Our analysis differs from those previous observations by investigating convergent mutations across V alleles in a population with multiple potential specificities, suggesting that convergent mutations may be rare overall when considering a whole pathogen."

5) Do the authors predict a similar relationship for usage of LC V genes?

Response: In principle, our theoretical reasoning and simulations apply to other germline gene sets we have not studied experimentally, such as light-chain variable genes.

Reviewer #2:

1. Overlap estimates may benefit from a Morisita's horn overlap index analysis.
2. In Figure 4 panel B, figures S9,S10,S12,S18 panel B, and S20 panel B. Legibility could be improved by replacing the numbers along the axis with the V Gene names which are overlapping in the current figures.

Response: Thank you for the suggestion. After careful consideration, we have tentatively concluded that our results already convey what the Morisita-Horn index might tell us – that the probability that two sequences drawn from different mice have the same V allele decreases over

time. As for the suggestion on the axis labels, we think it would be challenging because the overlap is partial and the order isn't exactly the same in different mice.

3. In 4B, plot titles are not clear, are these individual mice names?

Response: Yes, we apologize for the confusion. We changed the panel labels to indicate that each panel is a mouse.

Reviewer #3*: One overarching comment is that the main takeaway from both the simulations and the experiments is that the early response was indeed constrained by germline-based affinities but that as more and more mutations accumulated the response became less predictable and stereotypical however this early/late discussion is not made in the title, which I feel is misleading.*

Response: Thank you, we have changed the title.

Reviewer #4: *(No Response)*
